# Dioxepine-Peri-Annulated PMIs—Synthesis and Spectral and Sensing Properties

**DOI:** 10.3390/s23062902

**Published:** 2023-03-07

**Authors:** Yulian Zagranyarski, Diana Valentinova Cheshmedzhieva, Monika Mutovska, Anife Ahmedova, Stanimir Stoyanov

**Affiliations:** Faculty of Chemistry and Pharmacy, Sofia University, 1164 Sofia, Bulgaria

**Keywords:** perylene monoimides, seven membered ring annulation, DiAmSar, PET sensors, DFT

## Abstract

New perylene monoimide (PMI) derivatives bearing a seven-membered heterocycle and 1,8-diaminosarcophagine (DiAmSar) or N,N-dimethylaminoethyl chelator fragments were synthesized, and their spectroscopic properties in the absence and presence of metal cations were determined to evaluate their potential applications as PET optical sensors for such analytes. DFT and TDDFT calculations were employed to rationalize the observed effects.

## 1. Introduction

Metal ions play a substantial role in the functioning of every living organism. On the one hand, their presence as macro or microelements is essential for almost any biological process, such as intra- and intercellular communications, electron and proton transfer processes, photosynthesis, the regulation of nerve impulses, gas transfers, etc. [1]. On the other hand, depending on the absorbed dose, they may be a major threat to the health and life of living systems [2]. There are plenty of scientifically proven environmental and health effects of metals, their cations, and their complexes, as well as several myths about their toxicity [3], but it is inarguable that their qualitative and quantitative detection is of utmost importance [4]. One of the most versatile and promising techniques for detecting and quantifying cations of interest are fluorescent sensors [5], which are capable of changing their emissions upon specific binding with the target analytes [6]. Such fluorometric probes provide excellent sensitivity, fast responses [7], and are widely applied in both optical imaging and analytical sensing [8,9].

The rational design of optical sensors incorporates a fluorophore as an active signaling unit, which is covalently tethered to a receptor fragment through a spacer [10]. While the chelating moiety provides the selectivity, an appropriate mechanism for translating the chemical changes to a modulated signal from the reporter should be employed. Photoinduced electron transfer (PET) is one of the most popular such techniques since it can selectively switch the fluorescence emission on or off in the presence of analytes [11,12]. Various classes of light-active organic compounds were successfully used as fluorophores in the design of PET sensors—1,8-naphthalimides, benzanthrone, benzofurazan and xanthene derivatives, etc. [13,14,15,16,17,18]. One of the emerging classes of fluorophores used as signal fragments are perylene monoimides (PMIs) and diimides (PDIs) [19,20,21]. Due to their outstanding chemical, thermal, and photostability, they are among the most promising functional dyes [22,23] and have numerous applications in organic electronics [24], photovoltaics [25], membrane labeling [26], and OLEDs [27]. Due to the constant search for receptor fragments providing the best possible match for any specific fluorophore, various open-chain and cyclic chelators have been tested. A very promising macrobicyclic hexamine cage ligand named sarcophagine (Sar = 3,6,10,13,16,19-hexaaza-bicyclo[6.6.6]-eicosane) is very well known and widely used for developing ^64^Cu complexes for radiolabeling [28,29,30], but, to the best of our knowledge, it has never been used as a receptor in PET optical sensors. Diamino-sarcophagine (DiAmSar) was chosen as the most promising Sar derivative for this purpose, as it is relatively easy to chemically attach to different organic structures [31] and has been proven to be able to quench the intrinsic fluorescence of albumins [32].

The aim of this work is to synthesize the first PMI derivatives with seven membered dioxepine rings annulated to the *peri*-positions, as well as having DiAmSar or N,N-dimethylaminoethyl chelating moiety, and to investigate their photophysical properties in the absence and presence of metal cations to determine the PET sensing potential of the new compounds. To better evaluate the proposed PET sensor design, a fluorophore without a receptor was also prepared, and all the structures were investigated via density-functional theory (DFT) and time-dependent density-functional theory (TDDFT) methods to gain a better understanding of the relevant photophysical processes.

## 2. Materials and Methods

UV/VIS absorption spectra were recorded on a “Cary 5000 UV/VIS/NIR” spectrophotometer (Varian, Mulgrave, Australia). Fluorescence investigations were performed on a “Cary Eclipse” spectrophotometer (Varian, Australia). In all spectroscopic measurements, 1 cm path length synthetic quartz glass cells were used. The organic solvents (spectroscopic grade) used in this study were dichloromethane (DCM) and methanol (MeOH).

The relative fluorescence quantum yield (RQY) was calculated based on the absorption and fluorescence spectra in the same solvent mixture (DCM/MeOH 1:1 v/v), assuming 100% emission quantum yield for the reference compound in the series. The RQYs were calculated as the Grad_sample_/Grad_ref_ ratio (%), where Grad_sample_ and Grad_ref_ are the gradients (slopes) of the integrated emission band areas as a function of the absorbance (measured at five different concentrations) for the sample and reference compound, respectively. The RQYs are only used to compare the emissions of the studied compounds and should not be compared with other published fluorescence quantum yields.

To study the effect of the metal cations on the fluorescence intensity, a series of solutions were prepared in volumetric flasks by adding increasing amounts of stock metal cation solution (c = 10^−2^ mol l^−1^) to the same volume of the studied compound and then diluting the mixture to the same final volume. The following salts were used as sources of metal cations: AgNO_3_, BaCl_2_.2H_2_O, Cu(NO_3_)_2_.5H_2_O, FeCl_3_.6H_2_O, MgCl_2_.6H_2_O, Pb(NO_3_)_2_, SnCl_2_.2H_2_O, and Zn(NO_3_)_2_.4 H_2_O (Sigma-Aldrich, Darmstadt, Germany).

The NMR spectra were obtained using a Bruker DRX-250 spectrometer (Bruker, Karlsruhe, Germany) operating at 400 and 101 MHz for ^1^H and ^13^C, respectively, using a dual 5 mm probe head. DMSO-d_6_ and CDCl_3_ were used as solvents. Thin layer chromatographic (TLC) analysis was performed on silica gel plates (Macherey-Nagel F_60_ 254 40 × 80; 0.2 mm, Macherey-Nagel, Duren, Germany) using the solvent system n-hexane/dichloromethane as an eluent, unless otherwise stated.

### 2.1. Synthesis

#### 2.1.1. Synthesis of 1,8-Diamino-3,6,10,13,16,19-hexaazabicyclo-(6,6,6)eicosane (DiAmSar)

The bicyclic cage structure of DiAmSar was synthesized via four-step template synthesis following known procedures [33,34], and the synthetic steps are represented in the Supplementary Materials. Briefly, a Co(en)_3_Cl_3_ complex (en = ethylenediamine) was isolated first and used as a template in the following step, in which it was reacted with formaldehyde and nitromethane in an ice-cold basic medium. The template-directed condensation involves all six N-atoms in the octahedron’s vertices of the Co(en)_3_Cl_3_ complex, thereby forming the bicyclic cage structure of Co(diNO_2_Sar)Cl_3_ (diNO_2_Sar = dinitrosarcophagine, or 1,8-dinitro-3,6,10,13,16,19-hexaazabicyclo-(6,6,6)eicosane). The latter was precipitated via the addition of 6N HCl and isolated as orange crystals. Its reduction to the diamino-analogue Co(DiAmSar)Cl_3_ was performed under nitrogen with an excess of Zn powder and concentrated HCl. To avoid the isolation of the mixture of Co(III) and Co(II) complexes, of which the latter is not stable in air, the formed product was oxidized to Co(III) with H_2_O_2_. The resulting product mixture of differently protonated amines was separated by column chromatography with the cation exchange resin Dowex 50WX2 (Sigma-Aldrich, Darmstadt, Germany) to allow for the isolation of the doubly protonated Co(DiAmSarH_2_)Cl_5_ via elution with 3M HCl. Yellow crystals were isolated after the concentration of the eluate. The removal of the Co(III) ion from the bicyclic cage was performed with an excess of cobaltous cyanide (formed in situ) and sodium cyanide under nitrogen. The metal-free DiAmSar was extracted with boiling acetonitrile from the dry residue of the evaporated reaction mixture. White crystals were collected after recrystallization from a minimal amount of acetonitrile.

^1^H-NMR (δ (ppm), DMSO-d_6_): 2.61 (s, 6H); 2.62 (s, 6H); 3.09 (bs, 10H, NH, NH_2_).

^13^C-NMR (δ (ppm), DMSO-d_6_): 50.00; 55.34; 58.64.

All intermediate products were characterized by IR (in KBr) and ^1^H NMR, and the spectra are given in the Supplementary Materials.

#### 2.1.2. Synthesis of Dibutyl 9,10-Dibromo-1,6,7,12-tetrachloroperylene-3,4-dicarboxylate (**B2**)

To a suspension of crude 9,10-dibromo-1,6,7,12-tetrachloroperylene-3,4-anhydride [35] (**B1**) (5 mmol 3.09 g), potassium hydroxide (20 mmol, 1.32 g) in 100 mL of water and 0.5 mL of aliquat 336 was added. The mixture was stirred and refluxed for 30 min, and then butyl bromide (50 mmol, 5.40 mL) was added in one portion. The reaction mixture was refluxed for 2 h and was then cooled down to room temperature and extracted with dichloromethane. The crude product was purified via column chromatography using hexane/dichloromethane as the eluent on silica. It yielded 3.29 g (88%) as a yellow solid.

^1^H-NMR (δ (ppm), CDCl_3_): 1.00 (t, 6H, CH_3_, J = 7.4 Hz); 1.50 (m, 4H, CH_2_); 1.80 (m, 4H, CH_2_); 4.36 (m, 4H, CH_2_, J = 10.8 Hz, J = 6.8 Hz); 8.03 (s, 2H); 8.05 (s, 2H).

^13^C-NMR (δ (ppm), CDCl_3_): 13.92; 19.37; 30.71; 66.13; 121.44; 123.09; 125.01; 125.12; 127.55; 131.45; 130.28; 132.11; 132.70; 133.95; 134.21; 135.70; 136.86; 167.22.

#### 2.1.3. Synthesis of Dibutyl 3,4,15,16-Tetrachloronaphtho[2,3-b]peryleno[3,4-ef][1,4]dioxepine-1,18-dicarboxylate (**B3**)

A mixture of dibutyl 9,10-dibromo-1,6,7,12-tetrachloroperylene-3,4-dicarboxylate (**B2**) (4.0 mmol, 2.99 g), 2,3-dihydroxynaphthalene (5.0 mmol, 0.80 g), and potassium carbonate (10 mmol, 1.38 g) in 20 mL NMP was stirred and heated to 140 °C for 2 h. The mixture was cooled down to room temperature and poured into ice containing 2 mL of concentrated hydrochloric acid. The precipitate was filtered, washed with water, and dried. The crude product was purified via column chromatography using hexane/dichloromethane as the eluent on silica. This yielded 2.72 g (91%) as an orange solid.

^1^H-NMR (δ (ppm), CDCl3): 1.00 (t, 6H, CH_3_, J = 7.4 Hz); 1.50 (m, 4H, CH_2_); 1.80 (m, 4H, CH_2_); 4.35 (qt, 4H, CH_2_, J = 10.8 Hz, J = 6.8 Hz); 7.48 (m, 2H); 7.61 (s, 2H); 7.81 (m, 2H); 7.83 (s, 2H); 8.05 (s, 2H).

^13^C-NMR (δ (ppm), CDCl3): 13.93; 19.38; 30.73; 65.98; 115.18; 119.08; 119.45; 121.29; 123.37; 126.59; 127.46; 127.91; 129.46; 131.45; 131.92; 132.02; 134.65; 135.37; 135.59; 148.82; 153.11; 167.41.

Elemental analysis (Anal.) Calculated (calcd.) for C_40_H_28_Cl_4_O_6_: C, 64.36; H, 3.78; Found: C, 64.51; H, 3.99.

#### 2.1.4. Synthesis of Dibutyl 3,4,15,16-Tetrachloronaphtho[2,3-b]peryleno[3,4-ef][1,4]dioxepine-1,18-dicarboxylate (**B4**)

To a solution of **B3** (3.5 mmol, 2.61 g) in 50 mL of glacial acetic acid, 10 mL sulfuric acid (95–97%) was added. The mixture was stirred at 110 °C for 2 h. The mixture was cooled down to room temperature and poured into ice. The precipitate was filtered, washed with water, and dried. This yielded 2.09 g (97%) as a purple solid. The product was pure according to the TLC analysis and was used in the next steps without further purification.

#### 2.1.5. Synthesis of 5,6,17,18-Tetrachloro-2-(2,6-diisopropylphenyl)-1H-naphtho[2″,3″:2′,3′][1,4]dioxepino[5′,6′,7′:9,10]peryleno[3,4-cd]pyridine-1,3(2H)-dione (**1a**)

To a suspension of **B4** (1.0 mmol, 0.62 g) in a mixture of 25 mL of NMP and 5 mL of glacial acetic acid, 2,6-diisopropylaniline (4.0 mmol, 0.71 g) was added. The mixture was stirred at 150 °C for 8 h. After cooling to room temperature, 30 mL water was added and the precipitate was filtered, washed with methanol, and dried. The product was purified via column chromatography using hexane/dichloromethane as the eluent on silica. This yielded 0.63 g (81%) as a purple solid.

^1^H-NMR (δ (ppm), CDCl_3_): 1.17 (d, 6H, CH_3_, J = 6.1 Hz); 1.19 (d, 6H, CH_3_, J = 6.0 Hz); 2.74 (hept, 2H, CH_,_ J = 6.8 Hz); 7.35 (d, 2H, J = 7.8 Hz); 7.51 (m, 3H); 7.71 (s, 2H); 7.84 (m, 2H); 7.87 (s, 2H); 8.66 (s, 2H).

^13^C-NMR (δ (ppm), CDCl_3_): 24.02; 29.21; 115.39; 119.00; 119.87; 120.68; 121.19; 123.81; 124.17; 126.63; 127.38; 129.81; 130.31; 131.37; 132.30; 132.86; 133.12; 135.30; 136.49; 145.66; 148.42; 148.51; 153.92; 162.81.

Anal. calcd. C_44_H_27_Cl_4_NO_4_: C, 68.15; H, 3.51; N, 1.81; Found: C, 68.33; H, 3.38; N, 1.90.

#### 2.1.6. Synthesis of 5,6,17,18-Tetrachloro-2-(2-(dimethylamino)ethyl)-1H-naphtho[2″,3″:2′,3′][1,4]dioxepino-[5′,6′,7′:9,10]peryleno[3,4-cd]pyridine-1,3(2H)-dione (**1b**)

To a suspension of **B4** (1.0 mmol, 0.62 g) in a mixture of 25 mL of NMP and 5 mL of glacial acetic acid, N^1^,N^1^-dimethylethylenediamine (4.0 mmol, 0.35 g) was added. The mixture was stirred at 120 °C for 2 h. After cooling to room temperature, 30 mL of water was added and the precipitate was filtered, washed with methanol, and dried. This yielded 0.65 g (95%).

^1^H-NMR (δ (ppm), DMSO-d_6_): 2.35 (s, 6H, NCH_3_); 2.66 (m, 2H, CH_2_); 4.34 (m, 2H, CH_2_); 7.49 (m, 2H); 7.66 (s, 2H); 7.82 (m, 2H); 7.84 (s, 2H); 8.58 (s, 2H).

^13^C-NMR (δ (ppm), DMSO-d_6_): 38.55; 45.95; 57.16; 115.43; 119.10; 119.92; 120.81; 121.37; 123.36; 126.72; 127.49; 130.11; 131.48; 132.23; 132.83; 132.87; 135.32; 136.48; 148.64; 153.92; 162.93.

Anal. calcd. C_36_H_20_Cl_4_N_2_O_4_: C, 63.00; H, 2.94; N, 4.08; Found: C, 63.21; H, 3.07; N, 4.19.

#### 2.1.7. Synthesis of 2-(8-Amino-3,6,10,13,16,19-hexaazabicyclo[6.6.6]icosan-1-yl)-5,6,17,18-tetrachloro-1H-naphtho[2″,3″:2′,3′][1,4]dioxepino[5′,6′,7′:9,10]peryleno[3,4-cd]pyridine-1,3(2H)-dione (**1c**)

To a suspension of **B4** (1.0 mmol, 0.62 g) in a mixture of 25 mL of NMP and 5 mL of glacial acetic acid, DiAmSar (2 mmol, 0.63 g) was added. The mixture was stirred at 150 °C for 12 h. After cooling to room temperature, 30 mL of water was added and the precipitate was filtered, washed with water, and dried. The product was purified via column chromatography using dichloromethane/methanol as the eluent on silica. This yielded 0.30 g (33%) as a purple solid.

^1^H-NMR (δ (ppm), DMSO-d_6_): 1.25 (m, 6H, CH_2_); 1.82 (m, 6H, CH_2_); 2.34 (t, 6H, CH_2_, J = 7.3 Hz); 2.85 (t, 6H, CH_2_, J = 7.5 Hz); 7.22-7.56 (m, 6H); 7.98 (bs, 2H); 8.14 (bs, 2H); 7.84 (s, 2H); 9.03 (bs, 6H, NH); 12.23 (bs, 2H, NH_2_).

Anal. calcd. C_46_H_42_Cl_4_N_8_O_4_: C, 60.54; H, 4.64; N, 12.28; Found: C, 60.30; H, 4.81; N, 12.07.

### 2.2. Computational Details

The geometry optimization and photophysical properties of the compounds were modeled with the G16 software package [36]. The optimization of the ground and excited state geometry for **1a–c** was performed within the DFT [37] and TDDFT [38] formalisms, respectively. The theoretical computations were carried out using B3LYP/6-31G(d,p) and PBE0/6-311+G(2d,p) levels of theory. The vibrational frequencies were evaluated for each structure at the same method/basis to verify that the structures are indeed a minimum of the potential energy surface, and no imaginary frequency was found.

The absorption wavelengths were determined by TDDFT calculations of vertical excitations. The absorption spectra of **1a–c** were simulated by TDDFT using the same functionals and basis sets. Twelve excited singlet states were studied. The lowest energy transition with non-zero oscillator strength is considered for each molecule. To simulate the fluorescence, the optimization of the excited state corresponding to the transition of interest was performed at TDDFT, starting from the ground state geometry. Calculations of the vibrational frequencies and the absence of imaginary frequencies confirm that the excited state geometries are minimum on the potential energy surface. The fluorescence electronic transitions were calculated as vertical de-excitations based on the optimized geometries of the excited state. A detailed description of the procedure can be found elsewhere [39].

The solvent effects were examined at each step by means of PCM formalism [40]. All the computations were performed in methanol as the solvent.

## 3. Results and Discussion

### 3.1. Synthesis of Compounds

**Scheme 1:**
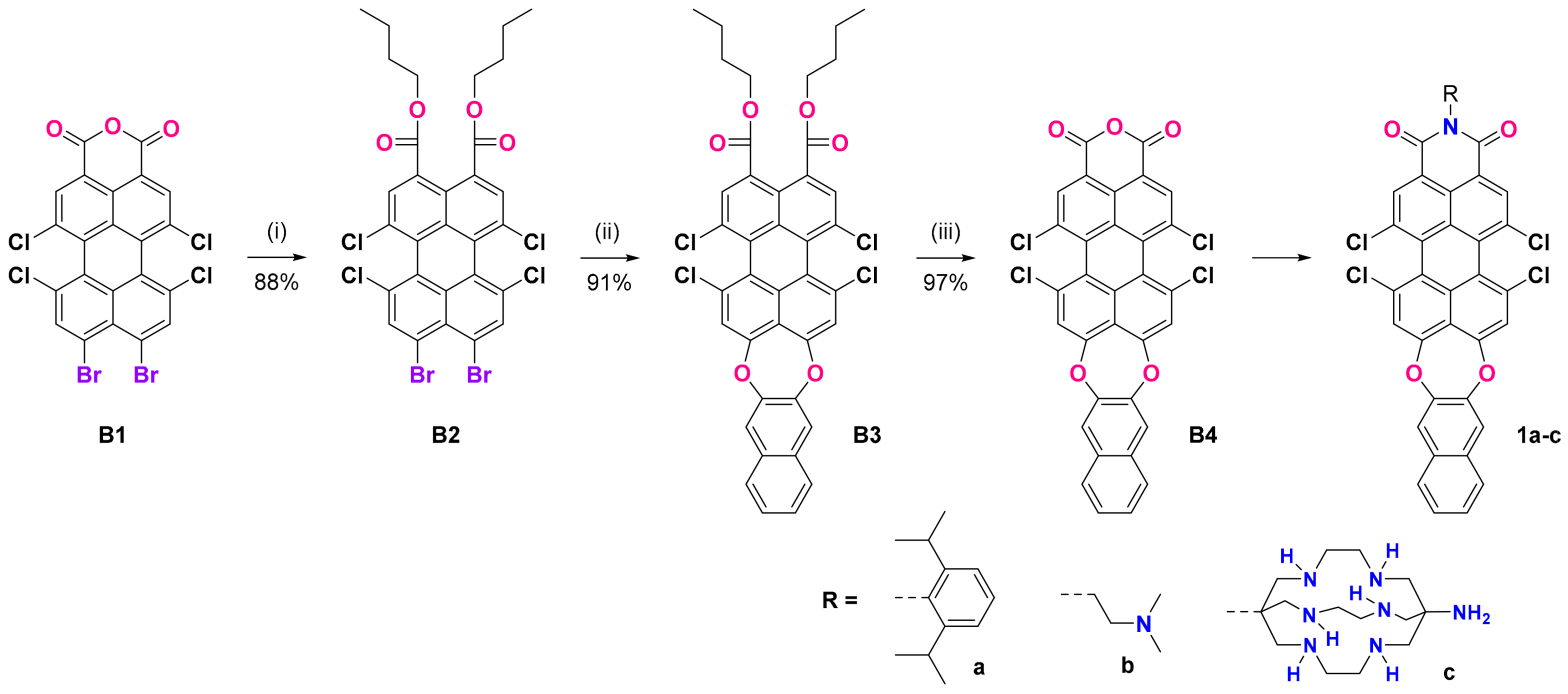
Preparation of N-substituted dioxepino PMIs **1a–c**: (i) KOH, aliquat 336, water, and then BuBr, reflux, 2 h; (ii) 2,3-dihydroxynaphthalene, NMP, K_2_CO_3_, 150 °C, 2 h; (iii) H_2_SO_4_/CH_3_COOH, 110 °C, 2 h.

One of our synthetic goals was to obtain new PMI derivatives with an expanded heterocyclic system by annulating a new 7-membered 1,4-dioxepine ring at the peri position of the perylene core. A suitable building block was 9,10-dibromo-6,7,12,13-tetrachloroperylene monoanhydride (**B1**), which can be easily obtained in gram scale according to the procedure published by Zagranyarski et al. [41]. We intended to use 2,3-dihydroxynaphtalene to substitute both Br-atoms in basic media (NMP, K_2_CO_3_), but the anhydride was not reactive enough in such Nucleophilic Aromatic Substitution (S_n_Ar) reactions. One possible approach to overcome this was to perform the imidization first, which is usually conducted with a bulky amine to prevent stacking, thus improving the solubility and making the corresponding imides easier to purify via column chromatography. In this way, we were able to obtain **1a** in two steps with a 53% overall yield (Supplementary Materials, Figure S3), thus proving that ring extension via S_n_Ar with suitable dihydroxy arenes is possible. Unfortunately, this “direct” path towards PMIs with benzo or naphtho-dioxepine rings fused at positions 9 and 10 (*peri*-annulated) has its limitations: (i) all our efforts to use an amine with chelating properties (*vide infra*) led to no reaction or complex reaction mixtures, from which the target compounds were impossible to isolate and purify; (ii) it is practically impossible to hydrolyze **1a** to the corresponding anhydride (**B4**, Scheme 1), which would allow us to introduce the target receptor fragments. Therefore, we decided to employ the corresponding diester derivatives, which had the following advantages: (i) the opening of the anhydride cycle can be performed easily in basic or acidic media—our method of choice was saponification and then alkylation with 1-bromobutane; (ii) the 9,10-dibromodiester **B2** is compatible with the key ring extension S_n_Ar step, and we were able to substitute both peri-bromine atoms, thus forming the new seven-membered cycle with a very good yield; (iii) the resulting dioxepino-diester **B3** is of good solubility, can be easily purified, and can be practically quantitatively converted back to the corresponding anhydride **B4**; (iv) once the extended heterocyclic system is formed, a variety of amines can be used for the imidization of **B4**, making it a valuable building block for rylene chemistry.

The compounds **1a–c** were synthesized with different substituents at the imide N atom. The one with 2,6-diisopropylphenyl (**1a**) had no chelating fragments and was prepared to investigate the intrinsic photophysical properties of the new fluorophore. Compound **1b** bears a N,N-dimethylaminoethyl receptor fragment, which has been tested in our previous investigations with naphthalimides [42,43]. In contrast, in the case of **1c**, we used the bulky bicyclic sarcophagine (DiAmSar), which is known as an excellent chelator for many metal ions. The DiAmSar was obtained in a four-step template synthesis, which employs the template effect of a metal ion to ensure a high yield and the isolation of the unique product with a bicyclic (cage-like) structure that would otherwise be difficult to obtain in a metal-free organic synthesis. Starting from a simple CoCl_3_ salt, a stable octahedral complex with ethylenediamine (en) can be readily obtained with a high yield and purity. Using it as a substrate in the following one-pot condensation reaction with formaldehyde and nitromethane leads to the formation of the bicyclic structure stabilized by the Co(III) present in its core, Co(diNO_2_Sar)Cl_3_. As the Co(III) ions are known for their inertness, the formed complex and its reduced analogue Co(diNO_2_Sar)Cl_3_ are very stable, even in a highly acidic medium. Therefore, the removal of the cobalt ions is the most elaborate step, but several procedures are available in the literature [33,34], of which we employed the use of an excess of cyanide that first reduces the Co(III) to the more labile Co(II) complex. The Co(II) ion can more easily be removed from the cage via the formation of stable cobaltous cyanide complexes. The unambiguous proof that the metal ion is removed from the cage is the color of the isolated sarcophagine, which is white, in contrast to the intensely colored cobalt complexes in the preceding steps, and the absence of Co complex-related signals in the NMR spectra. The isolated white crystals of DiAmSar were used in the synthesis of **1c**.

### 3.2. Spectral Properties

**Figure 1:**
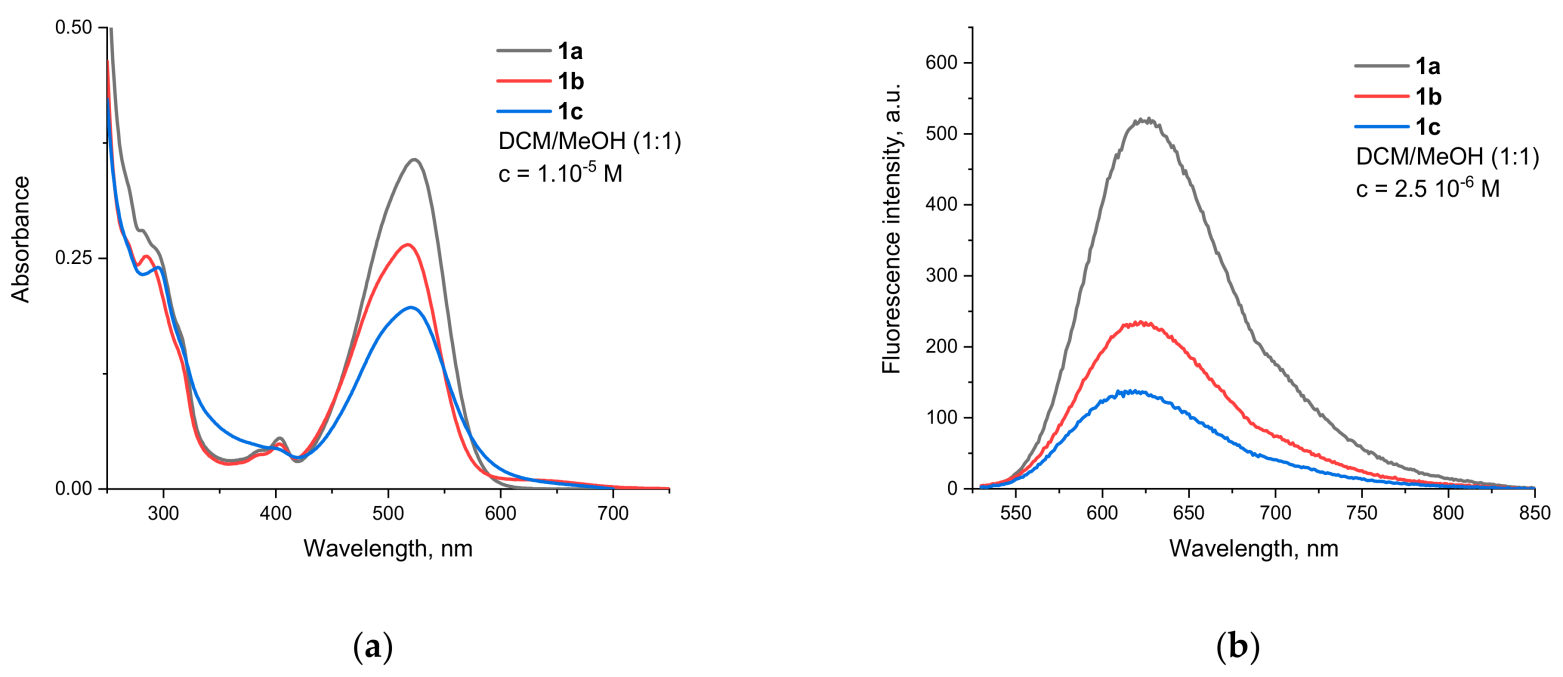
Spectra of compounds **1a–c** in DCM/MeOH 1:1 (v/v). (**a**) Absorption. (**b**) Fluorescence (excitation wavelength 520 nm).

Compounds **1a–c** show broad absorption bands in the 425–600 nm region, with maxima around 520 nm and a visible shoulder at the shorter wavelength side (Figure 1a). This is typical for rylene imides with two electron donating groups at the peri-positions due to the two different polarization modes in the formed donor–π–acceptor system [44].

Since the additional dioxepine cycle fused at positions 9 and 10 is not aromatic and does not lead to the extension of the perylene π-system, both oxygens should be considered auxochromes. Their donating capabilities are weakened due to their partial conjugation with the naphthalene unit in the opposite direction, but they still determine the intramolecular charge-transfer (ICT) nature of the observed transition. It should be noted that similar substitution patterns in which the two O-atoms are part of a seven membered cycle is not known from the literature. For similar structures, with two separate aryloxy substituents, Sahoo et al. reported a *λ_max_* of 540 and 590 nm for absorption and emission, respectively [45]. As Table 1 shows, the absorption bands of the compounds investigated in this work are slightly hypsochromically shifted, which can be attributed to the combined influence of several factors (vide infra).

The fluorescence emission spectra of compounds **1a–c** are shown on Figure 1b. All of them emit in the orange-red part of the spectrum and have maxima of around 620 nm. The bands are bathochromically shifted in comparison to the aforementioned diaryloxy-substituted PMIs, leading to Stokes shifts almost twice as big at around 3000 cm^−1^. This is an indication that substantial conformational changes take place upon the relaxation of the Franck–Condon to the locally excited (LE) emissive state.

**Figure 2:**
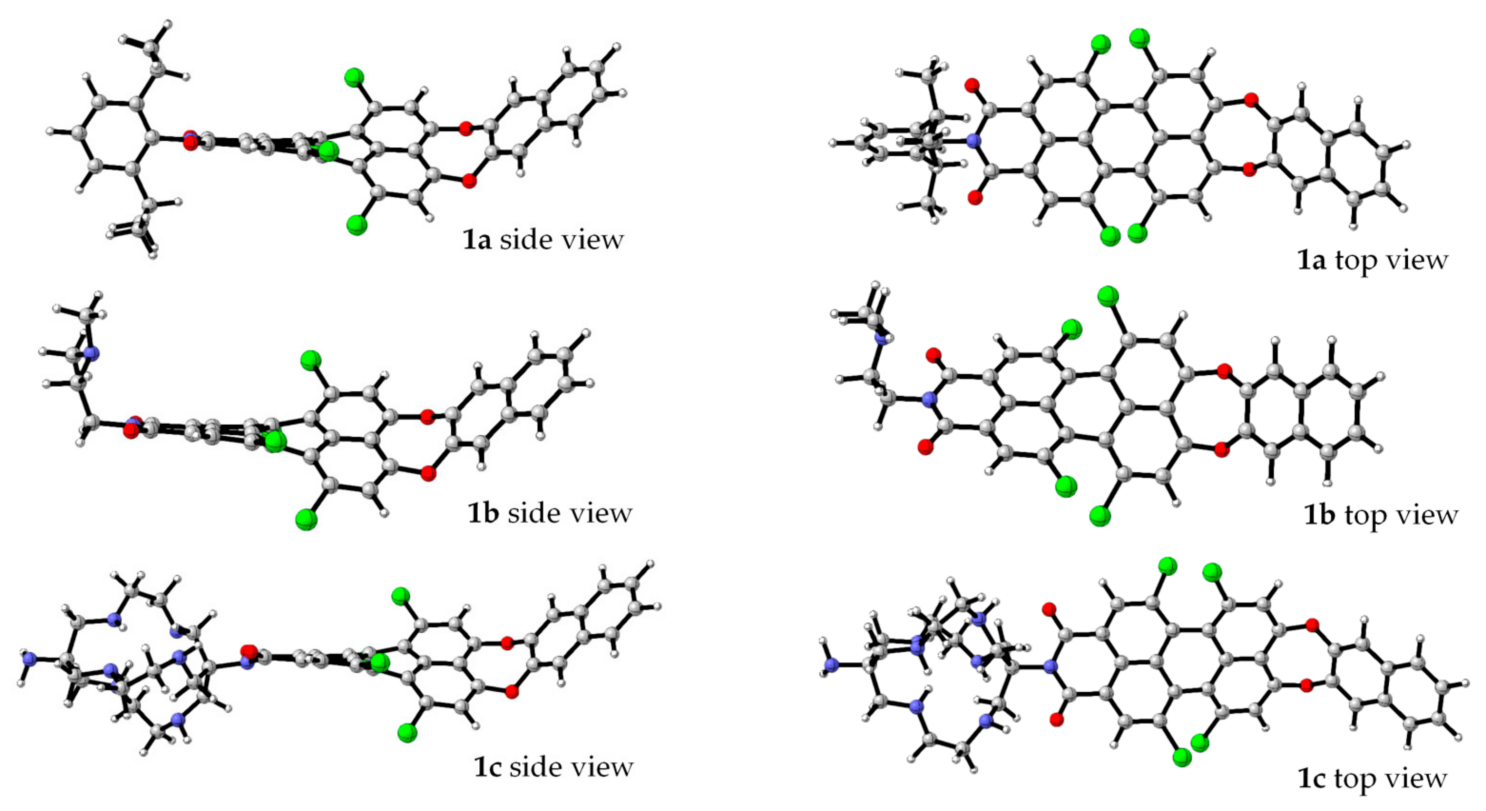
B3LYP/6-31G(d,p) optimized geometries of **1a–c** in methanol [46].

To clarify the nature of the electronic transitions and the influence of the N-substituents in the PMIs on the geometry and spectral properties of the studied compounds, DFT and TDDFT calculations were performed. The B3LYP/6-31G(d,p) optimized structures of **1a–c** in solvent methanol are presented in Figure 2. The gas-phase optimized molecular geometry of unsubstituted PMI is planar [40]. The bulky chlorine substituents in the bay positions result in the twisting of the molecular geometry to avoid steric hindrance. Therefore, the geometry is not planar, and the π–π interaction is weaker. The calculated (B3LYP/6-31G(d,p)) dihedral angles between the two naphthalene rings in the perylene moiety are rather high (37.5° for **1a** and 37.4° and 37.8° for **1b** and **1c**, respectively) and are consistent with the X-ray data for tetra-substituted PMIs [41].

It is known that electron-donor (EDG) substituents at the bay position cause a bathochromic shift of the longest-wavelength electronic transition in the compounds, while electron-withdrawing substituents (EWG) lead to a hypsochromic shift [47]. It can be concluded that the combination of the core twisting, fixed bended conformation at the donor part and the EWG at the bay position define the observed absorption maxima and are in line with the hypsochromic shift.

The theoretical results for the absorption transition energies obtained with the two functionals (B3LYP, and PBE0) and two basis sets (6-31G(d,p) and 6-311+G(2d,p)) in methanol solution for the three substituted PMIs are shown in Table 2. Substitution at the imide nitrogen atom usually improves the solubility of the compounds and modulates their aggregation properties in the solid state but has a very weak effect on their spectral properties, as can be seen from the experimental and theoretical data. Both combinations of functional and basis sets used for the spectral calculations proved to be suitable for the studied substituted PMIs. The transitions are simulated with good accuracy by the theoretical calculations. PBE0 gives better results, and the difference between the calculated and measured transitions is 0.08 eV at most.

**Figure 3:**
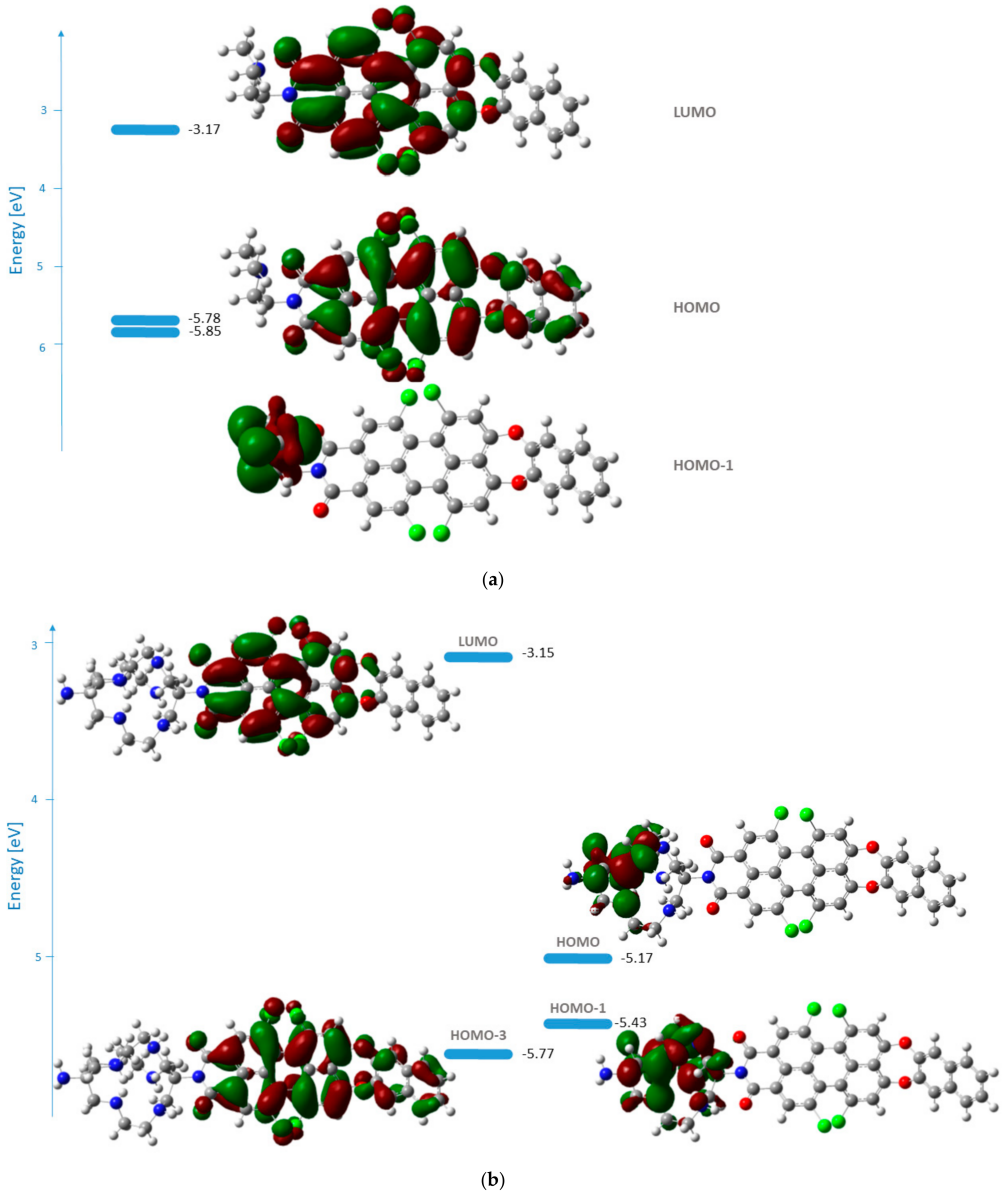
Energies and shape representations of the ground state orbitals in methanol from TDB3LYP/6-31G(d,p) computations: (**a**) HOMO−1, HOMO, and LUMO of **1b**; (**b**) HOMO−3, HOMO−1, HOMO, and LUMO of **1c**.

The frontier molecular orbitals for **1b** and **1c** in methanol are represented in Figure 3. The two functionals applied describe the orbitals’ shape in an analogous way.

The HOMO of **1b** is delocalized over the perylene core and the naphthodioxepine moiety (the donor), while LUMO covers the acceptor part of the molecule. The orbitals’ shape indicates that an effective intramolecular charge transfer (ICT) occurs from the donor group toward the imide moiety. Hence, the HOMO→LUMO transition for **1b** can be classified as an ICT transition with contributions from the aryloxy substituents. In the case of **1c**, the transition with the highest oscillator strength that corresponds to the one in the experimental spectrum arises from HOMO−3→LUMO. In this case, the nature of the transition is also ICT.

**Figure 4:**
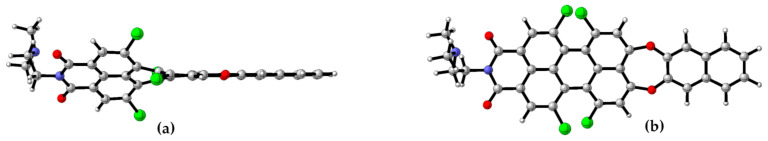
TDPBE0/6-311+G(2d,p)-optimized excited state geometry (S_1_) of **1b** in methanol. (**a**) Side view. (**b**) Top view.

The excited state geometries for **1b** and **1c** were optimized at the PBE0/6-311+G(2d,p) level. For **1b**, the predicted fluorescence maximum in methanol is 1.85 eV (671 nm and oscillator strength f = 1.12), which is in good agreement with the experimental value (1.99 eV). The geometry of the excited state of **1b** is shown in Figure 4. The main difference in the optimized geometry for the ground S_0_ and the excited S_1_ states concerns the dioxepine substituent at the peri position. The donor part of the molecule becomes planar in its excited state.

### 3.3. PET Design Evaluation

**Figure 5:**
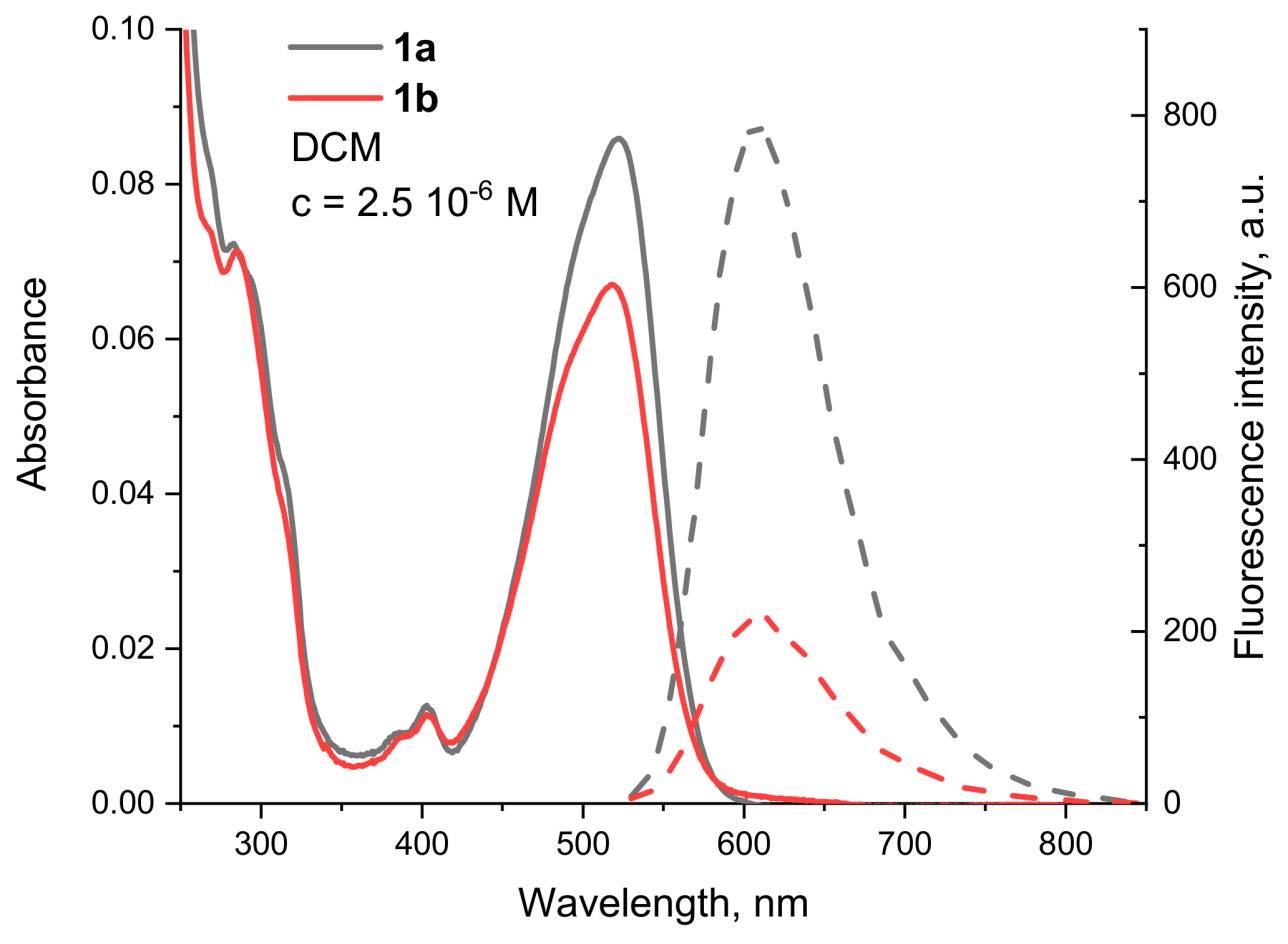
Absorption (solid line) and fluorescence (dashed line) spectra of **1a** (black) and **1b** (red) in DCM, c = 2.5 10^−6^ M.

Comparison of the fluorescence intensity of the sensor fragment containing compounds **1b,c** with that of the reference compound **1a** shows a decrease in the signal due to a PET from the nitrogen atoms that are two carbon atoms away from the imide nitrogen. This can best be illustrated by the spectra of compounds **1a** and **1b**, which are shown in Figure 5 and clearly show that the weaker fluorescence is not only due to the lower molar absorbance at the excitation wavelength.

From the relative quantum yields calculated in Table 1, the effect can be determined quantitatively. The most efficient fluorescence quenching was observed for **1c**, where the RQY was half that of **1a**. The emission of **1b** also clearly decreases due to the presence of the tertiary nitrogen of the dimethylaminoethyl group, with an RQY of 61%. These results suggest that the best match between the HOMO energies of the unbound donor and the fluorophore (condition for reductive PET) is found in **1c**. This was also confirmed by the calculated frontier molecular orbitals. For **1c** (Figure 3b), both HOMO and HOMO−1 are located at the DiAmSar fragment and can transfer an electron towards the HOMO−3, which is responsible for the ICT transition and is lower by 0.6 and 0.33 eV, respectively. This is consistent with a reductive PET process occurring for **1c**. For **1b**, the electron transfer should be from HOMO−1, which is located at the chelator, towards the HOMO, from where the electron is excited during the ICT transition (Figure 3a). The energy of the donor was found to be slightly lower than the acceptor, but the difference is only 0.07 eV, suggesting that PET is possible, although less favorable compared to **1c**. In general, it can be concluded that the design of the PET sensing system was successful, but the efficiency of the PET is not particularly great.

### 3.4. Influence of Metal Cations

**Figure 6:**
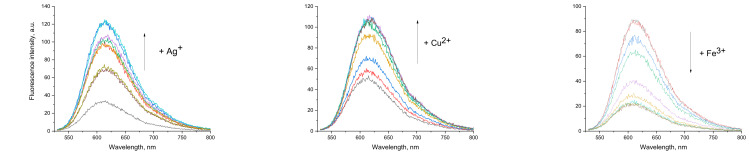
Illustrative examples of the observed changes in the fluorescence spectra of **1b** upon increasing the respective metal cation concentration.

The presence of analytes in the solution affects the emission of compound **1b**. While the absorption spectra remain the same when increasing the metal cations’ concentration, fluorescence intensity changes were observed. As is expected in PET sensing systems, blocking the electron transfer “turns on” the fluorescence without changing its wavelength. Fluorescence titration experiments were performed by varying the metal ion concentration from 0 to at least 2 times the concentration of the free ligand. Typical results are shown in Figure 6. The signal enhancement with increasing concentration of silver and copper ions is demonstrated. An interesting result is registered for Fe^3+^, where the intensity decreases (far right). A similar effect, but less pronounced, is also observed with Ba^2+^.

The PET sensors’ sensitivity can be quantified by the fluorescence enhancement (FE) factor, which is defined as the ratio of the maximum intensity achieved (when the signal reaches a plateau and the emission does not change with a further increase in the analyte’s concentration) to the initial intensity of the sample in the absence of analyte.

**Figure 7:**
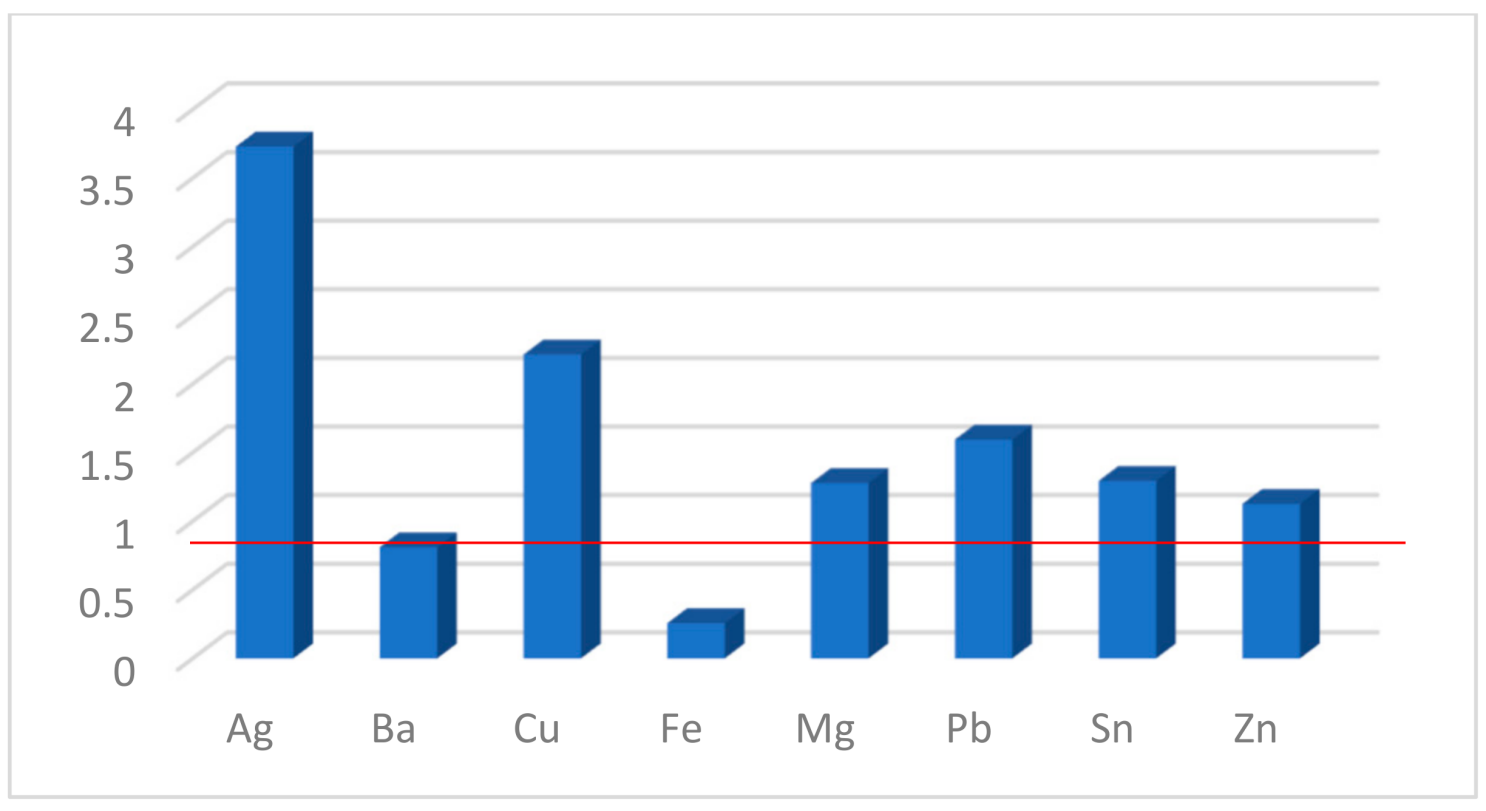
Fluorescence enhancement (FE) factors for **1b** in the presence of metal salts.

The FE factors calculated for compound **1b** are presented in Figure 7. As can be seen, the sensor has the greatest sensitivity to Ag^+^ and Cu^2+^. The values close to unity (the red line for FE = 1 shows no change whatsoever) for the remaining cations suggest some selectivity, most probably due to lower rates of complex formation in their presence. Titrations of **1c** did not lead to a change in the fluorescence intensity, from which it can be concluded that either there is no complex formation between the sarcophagine and the studied metal ions under these conditions (not very likely) or that the introduction of the analyte into the chelator cavity does not sufficiently engage all electron pairs at the nitrogen atoms in it and fails to block the PET.

**Figure 8:**
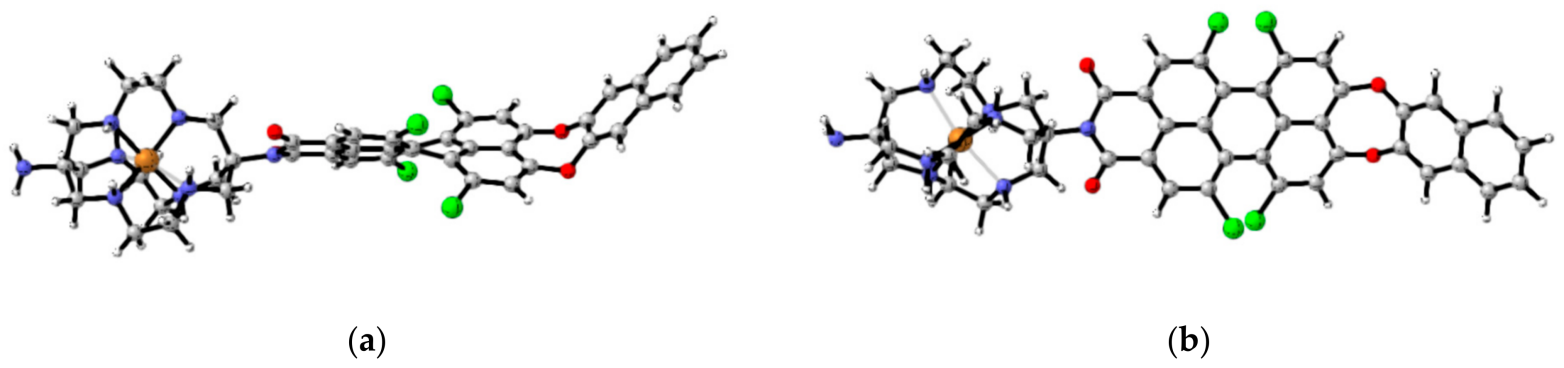
Optimized structure of the complex of **1c** with Cu^2+^ ions from B3LYP/6-31G(d,p) computations. (**a**) Side view. (**b**) Top view.

To gain a better understanding of the observed PET, even in the presence of a metal cation at the chelator, the geometry of **1c** complex with Cu^2+^ ions was studied theoretically at the B3LYP/6-31G(d,p) level of theory, and its structure is given in Figure 8. 

**Figure 9:**
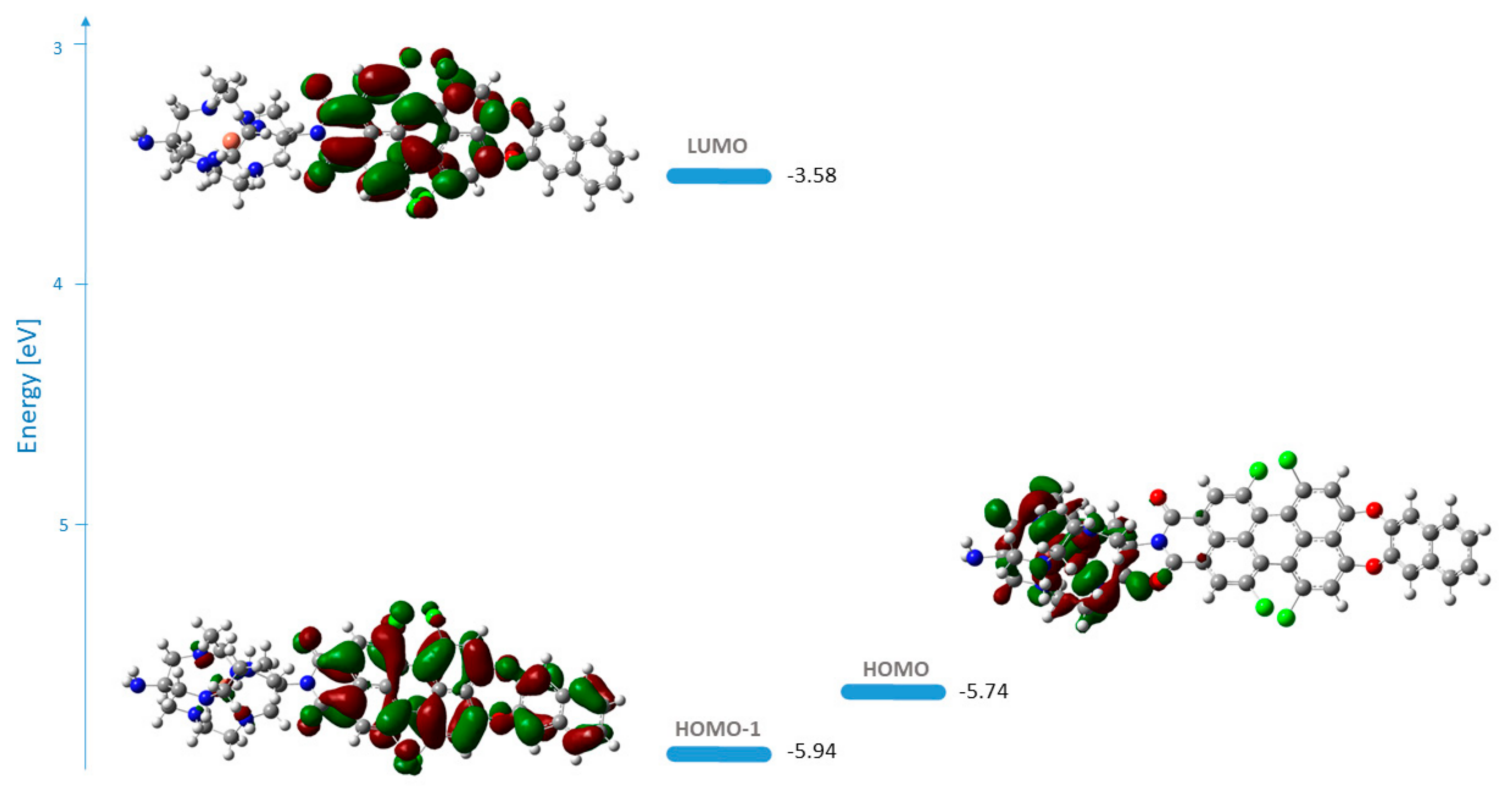
Energies and shape representation of HOMO−1, HOMO, and LUMO in the excited state (S_1_) of the complex of **1c** with Cu^2+^ ions in methanol from the TDB3LYP/6-31G(d,p) computations.

The S_1_-excited state of the complex was optimized at the same level of theory, and the energies and shape representation of the frontier molecular orbitals are given in Figure 9. The S_1_-excited state geometry optimization is necessary as the consideration of the MO in the ground state only is not sufficient to reliably predict the photoinduced electron transfer [14,48].

Fluorescence enhancement will only take place if the sarcophagine orbitals in the bound state lie lower than the fluorophore orbitals and the reductive PET process cannot occur. In the Cu^2+^ complex of **1c**, the intense transition localized on the fluorophore corresponds to excitation from the HOMO−1→LUMO transition (Figure 9). HOMO is located at the DiAmSar fragment, and an electron can be transferred towards HOMO−1, explaining its inability to block the PET and why **1c** cannot be used as a PET sensor.

We also optimized the geometry of the complex between **1b** and hydrated Cu^2+^ ions at the B3LYP/6-31G(d,p) level of theory (Figure S12 in Supplementary Materials). As can be seen, in this case the metal ion is coordinated with one of the N atoms and one O from the imide moiety of the dye, effectively engaging the electron lone pair at the tertiary nitrogen and blocking the PET.

## 4. Conclusions

Three new perylene monoimide (PMI) derivatives bearing a seven-membered 1,4-dioxepine ring fused at positions 9 and 10 (peri-annulated) were synthesized. An improved synthetic protocol utilizing diester derivatives was employed to maintain compatibility with the SnAr reaction while allowing for the easy purification and introduction of the target receptor fragments. Two Photoinduced Electron Transfer (PET) optical sensors bearing DiAmSar and N,N-dimethylaminoethyl chelator moieties were prepared along with a reference compound with classical 2,6-diisopropylphenyl substituents at the imide nitrogen. The intramolecular charge transfer (ICT) character of the transitions in the recorded absorption spectra was shown, and its efficiency was explained in terms of the molecular geometry and the overlapping of the corresponding molecular orbitals. Both receptor fragments were found to quench the intrinsic emission of the fluorophore, proving the successful design of the PET systems. The DiAmSar derivative **1c** showed a better match in the energies of the participating frontier molecular orbitals. Unfortunately, the complex formation does not engage the lone electron pairs in the chelator strongly enough to block the PET and the fluorescence signal does not change in the presence of metal cations. Another reason from a practical point of view is that cyclic chelators usually need additional time and energy to initiate complex formation. These demands are lower in the case of non-cyclic chelators. Therefore, a reasonable compromise between the kinetics of the complexation and the stability of the formed complexes is another concern in the design of a successful optical sensor for metal ions. Compound **1b** showed a fluorescence enhancement upon the addition of analytes, with selectivity towards Ag^+^ and Cu^2+^ with FE factors of 3.7 and 2.3, respectively.

## Figures and Tables

**Table 1:** Photophysical characteristics of the investigated compounds in mixed DCM/MeOH (1:1) solvent.

Compound	$\lambda_{max}^{ABS},\;\mathrm{nm}$	ε, l. mol^−1^ cm^−1^	$\lambda_{max}^{FLU},\;\mathrm{nm}$	$\Delta \nu_{St},\;{\mathrm{cm}}^{-1}$	RQY *, %
**1a**	523	35,700	625	3120	100 ^#^
**1b**	519	26,500	622	3191	61
**1c**	520	19,700	619	3076	48

* Relative fluorescence quantum yield measured towards the reference compound for the series. ^#^ reference compound for the series.

**Table 2:** Theoretically calculated absorption maxima (in eV), values of oscillator strength (f), and molecular orbital contributions to electronic transition in methanol for **1a–c** compared with experimental absorption maxima.

	B3LYP/6-31G(d,p)			PBE0/6-311+G(2d,p)		Experiment *
	*λmax* [eV]	f	Electronic Transition Contribution [%]	*λmax* [eV]	f	λmax [eV]
**1a**	2.26	0.6632	HOMO–LUMO 99.4	2.30	0.6862	2.37
**1b**	2.28	0.6224	HOMO–LUMO 99.4	2.31	0.6429	2.39
**1c**	2.19	0.6863	HOMO-4–LUMO 99.2	2.32	0.6928	2.38

* DCM/MeOH as a solvent.

## Supplementary Materials

The following supporting information can be downloaded at: https://www.mdpi.com/article/10.3390/s23062902/s1, Figure S1: Synthesis of DiAmSar.; Figure S2: Synthetic scheme for “direct” preparation of **1a**.; Figure S3. ^1^H NMR (a) and IR (b) spectra of Co(en)_3_Cl_3_.; Figure S4. ^1^H NMR (a), ^13^C NMR (b) and IR (c) spectra of [Co(diNO_2_Sar)]Cl_3_.; Figure S5. ^1^H NMR (a), ^13^C NMR (b) and IR (c) spectra of [Co(diAmSarH_2_)]Cl_5_.; Figure S6. ^1^H NMR (a) and ^13^C NMR (b) spectra of DiAmSar.; Figure S7. ^1^H NMR (a) and ^13^C NMR (b) spectra of B2.; Figure S8. ^1^H NMR (a) and ^13^C NMR (b) spectra of B3.; Figure S9. ^1^H NMR (a) and ^13^C NMR (b) spectra of **1a**; Figure S10. ^1^H NMR (a) and ^13^C NMR (b) spectra of **1b**.; Figure S11. ^1^H NMR spectrum of **1c**.; Figure S12. Optimized structure for the complex of **1b** with hydrated Cu^2+^ ion from B3LYP/6-31G(d,p) computations.; Table S1. Cartesian coordinates, electronic energies in Hartree, and the number of imaginary frequencies for the B3LYP/6-31G(d,p) fully-optimized geometries of the ground state for dyes **1a–c** in methanol and complexes of dyes **1b,c** with Cu^2+^ ions.; Table S2. Cartesian coordinates, electronic energies in Hartree, and the number of imaginary frequencies for the B3LYP/6-31G(d,p) fully optimized geometry of the excited state for the complex of dye **1c** with Cu^2+^ ions.; Table S3. Cartesian coordinates, electronic energies in Hartree, and the number of imaginary frequencies for the optimized geometry of the excited state for dye **1b** in methanol at the PBE1PBE/6-311+G(2d,p) level of theory.
