# Supplementary material for: Dioxepine-Peri-Annulated PMIs—Synthesis and Spectral and Sensing Properties
Source: Sensors (Basel). 2023 Mar 7;23(6):2902. doi: 10.3390/s23062902 (PMC10058915; doi:10.3390/s23062902)
Supplement: Supplementary file 1 [file sensors-23-02902-s001.zip › sensors-2210166-supplementary.pdf]

sensors

Supplementary materials

Dioxepine-Peri-Annulated PMIs—Synthesis and Spectral and Sensing Properties

Yulian Zagranyarski, Diana Valentinova Cheshmedzhieva, Monika Mutovska, Anife Ahmedova and Stanimir Stoyanov *

Faculty of Chemistry and Pharmacy, Sofia University, 1164 Sofia, Bulgaria
* Correspondence: sstoyanov@chem.uni-sofia.bg; Tel: +359-2-8161329

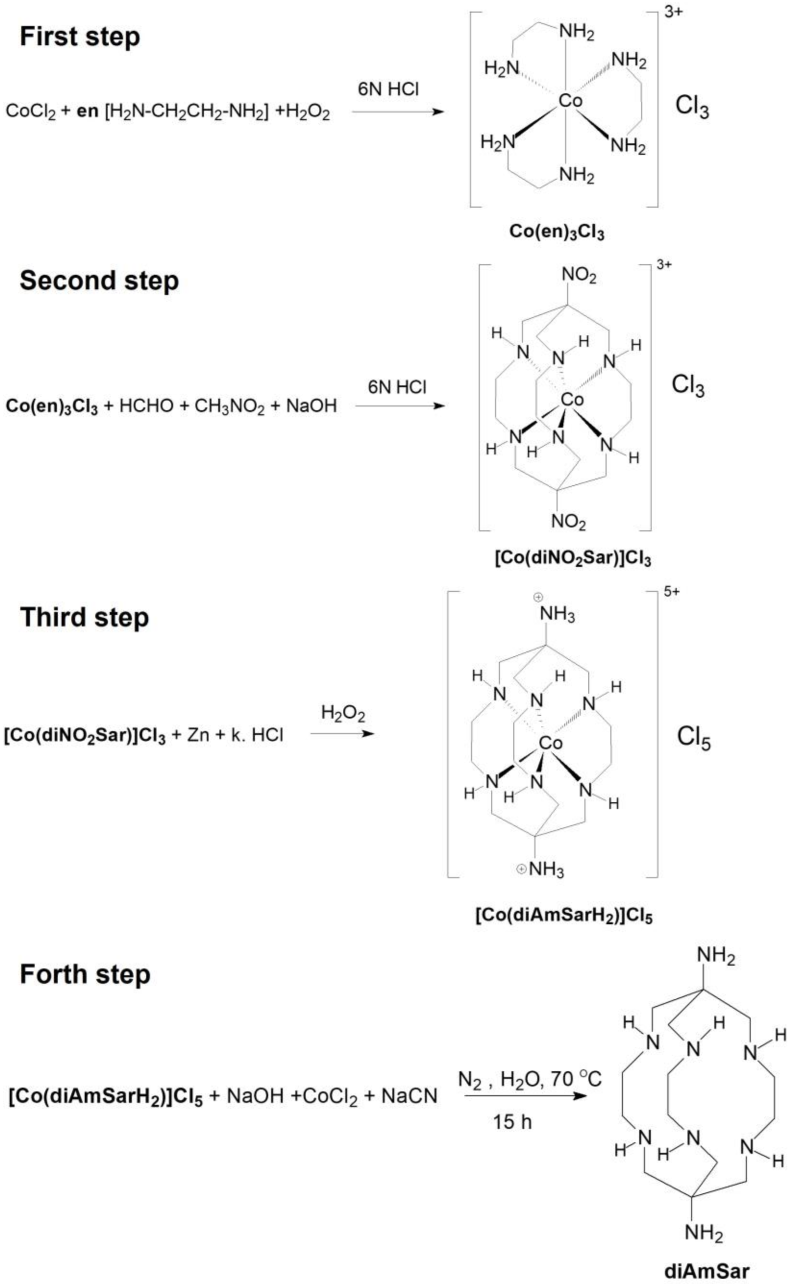

Figure S1. Synthesis of DiAmSar.

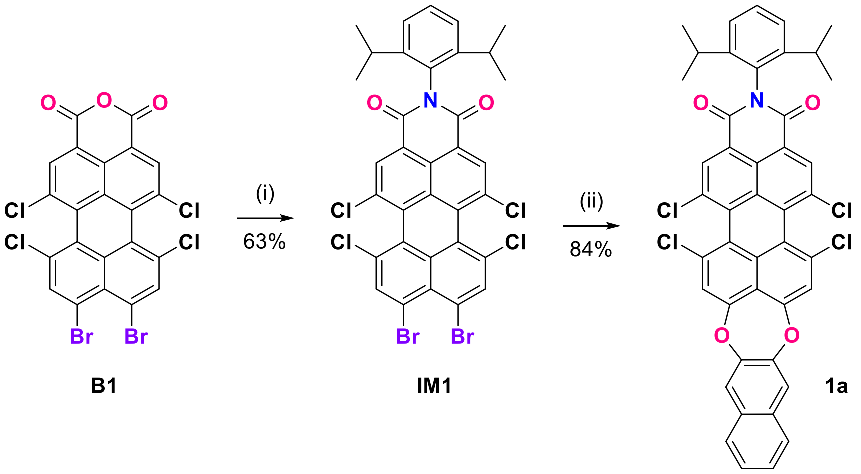

Figure S2. Synthetic scheme for "direct" preparation of **1a**: (i) 2,6-diisopropylaniline, NMP/CH_3COOH, 130°C, 8h; (ii) 2,3-dihydroxynaphthalene, NMP, K_2CO_3, 150°C, 2h.

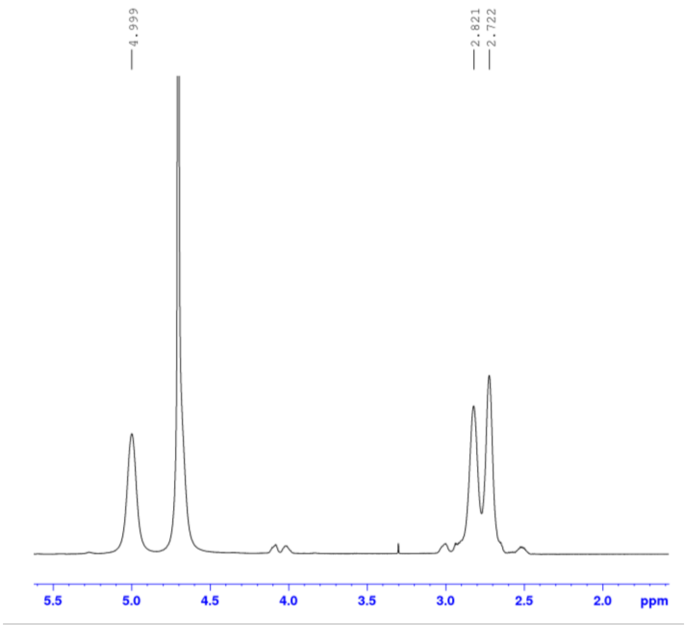

(**a**)

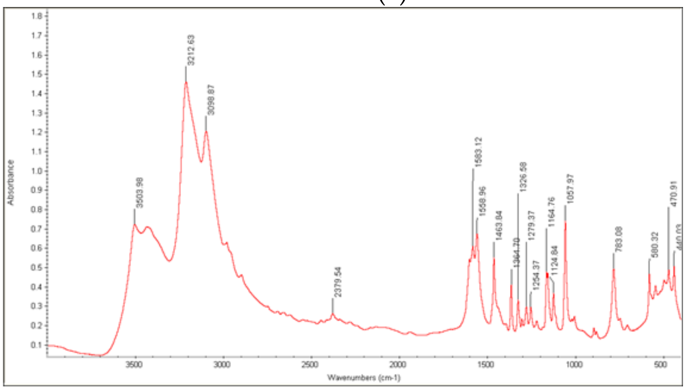

(**b**)

Figure S3. ^{1}H NMR (a) and IR (b) spectra of $Co(en)_3Cl_3$.

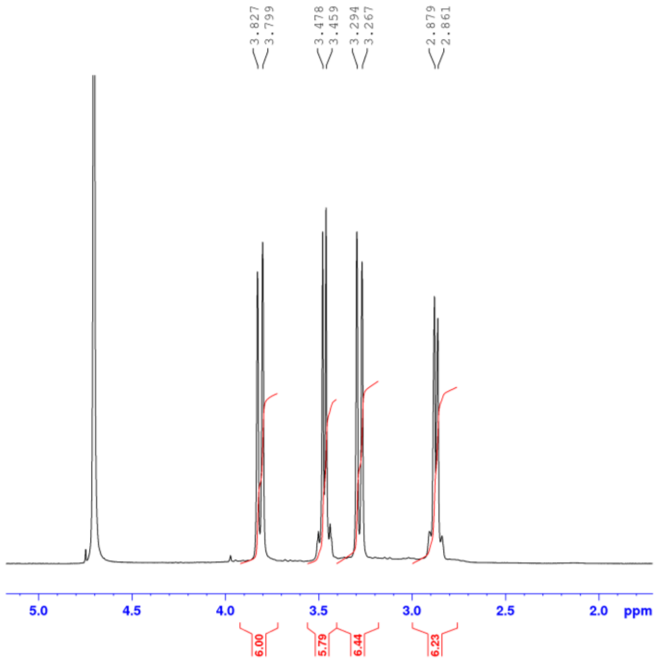

(**a**)

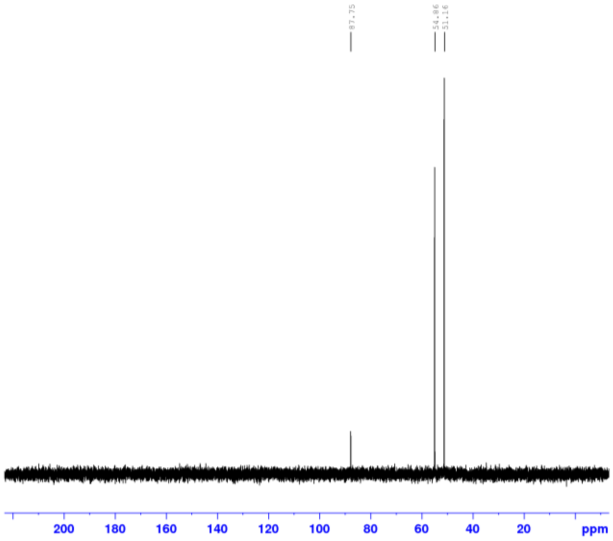

(**b**)

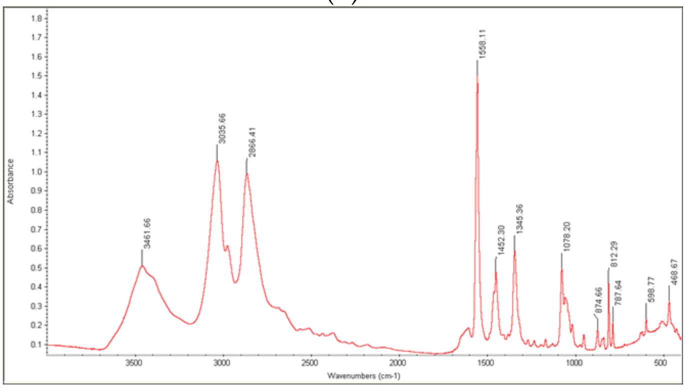

(c)

Figure S4. ^{1}H NMR (a), ^{13}C NMR (b) and IR (c) spectra of $[Co(diNO_2Sar)]Cl_3$.

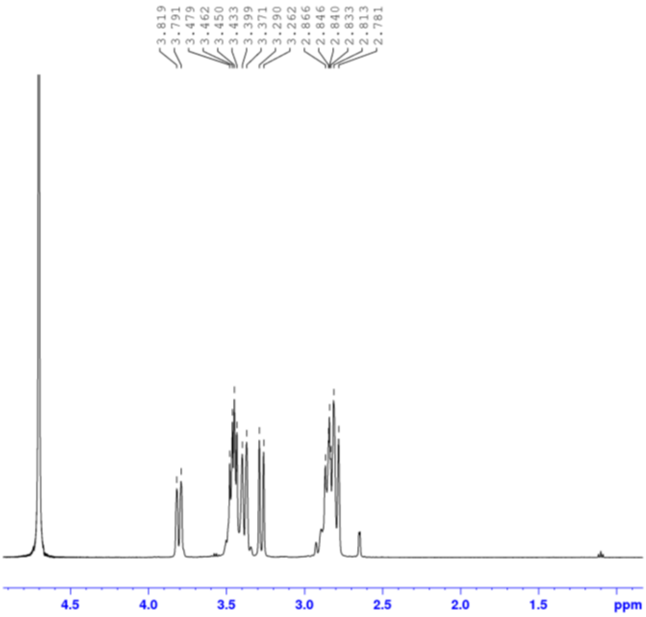

(**a**)

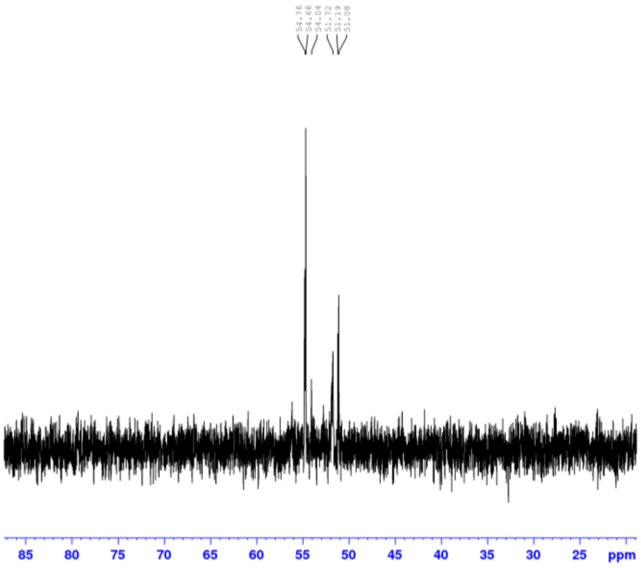

(**b**)

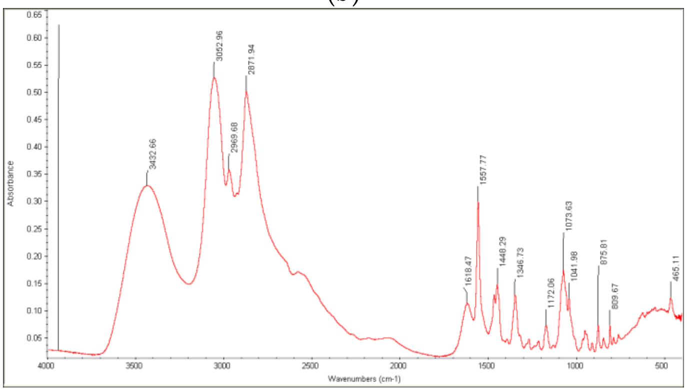

(c)

Figure S5. ^{1}H NMR (a), ^{13}C NMR (b) and IR (c) spectra of $[Co(diAmSarH_2)]Cl_5$.

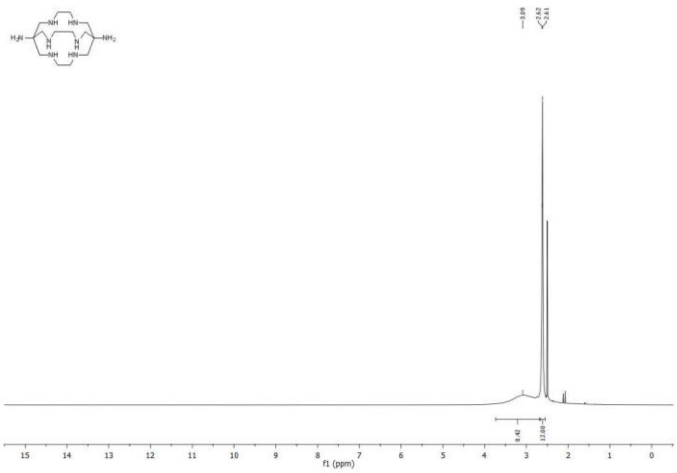

(**a**)

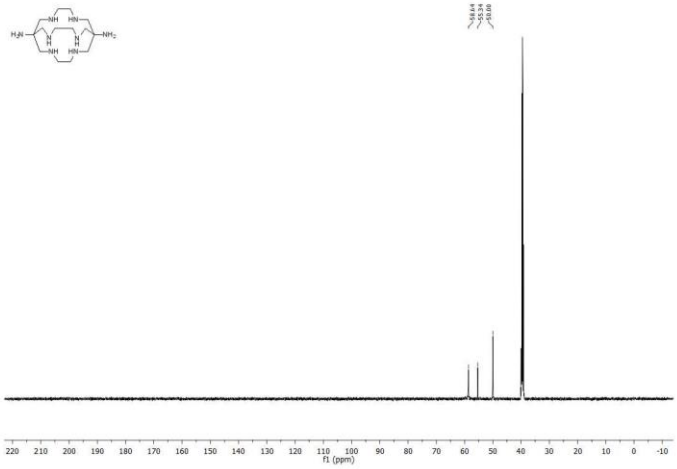

(**b**)

Figure S6. ^{1}H NMR (a) and ^{13}C NMR (b) spectra of DiAmSar.

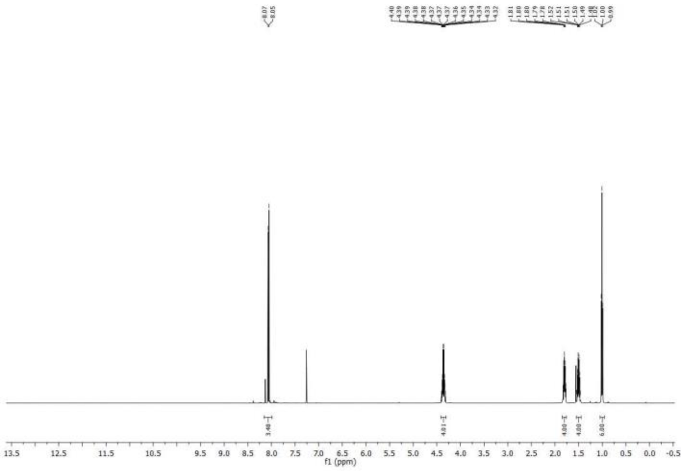

(**a**)

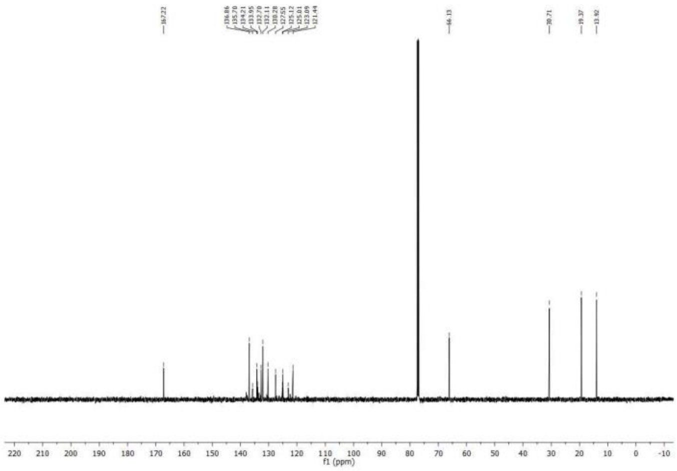

(**b**)

Figure S7. ^{1}H NMR (a) and ^{13}C NMR (b) spectra of **B2**.

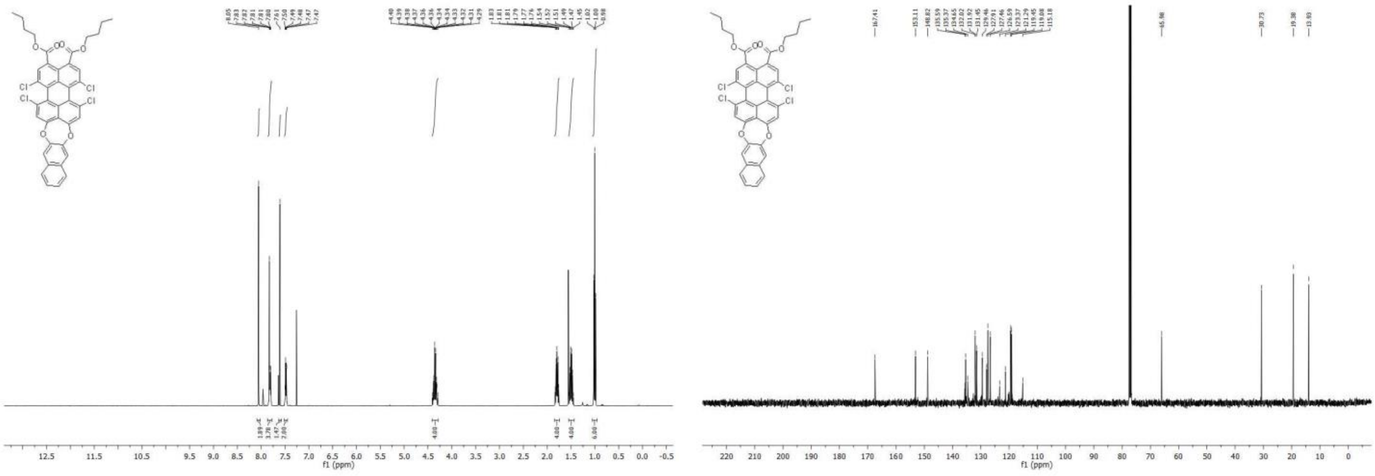

(**a**) (**b**)

Figure S8. ^{1}H NMR (a) and ^{13}C NMR (b) spectra of **B3**.

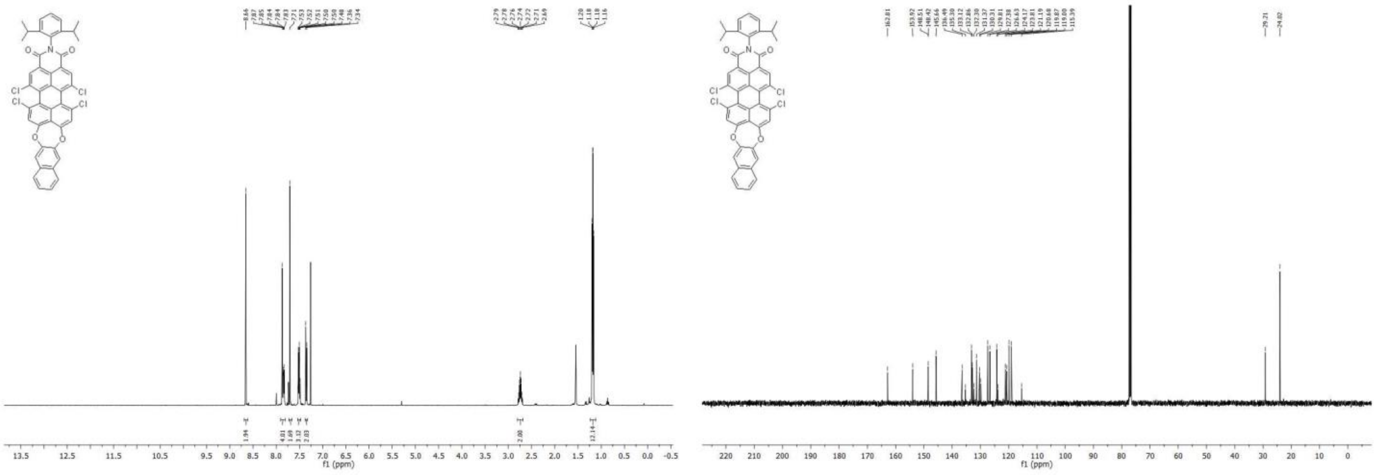

(**a**) (**b**)

Figure S9. ^{1}H NMR (a) and ^{13}C NMR (b) spectra of **1a**.

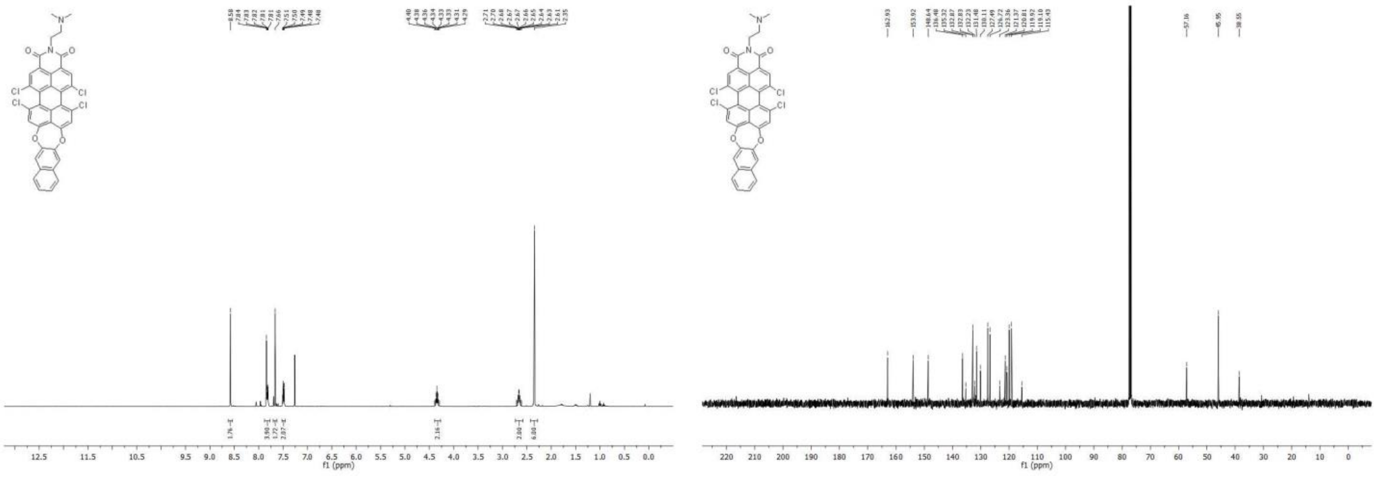

(**a**) (**b**)

Figure S10. ^{1}H NMR (a) and ^{13}C NMR (b) spectra of **1b**.

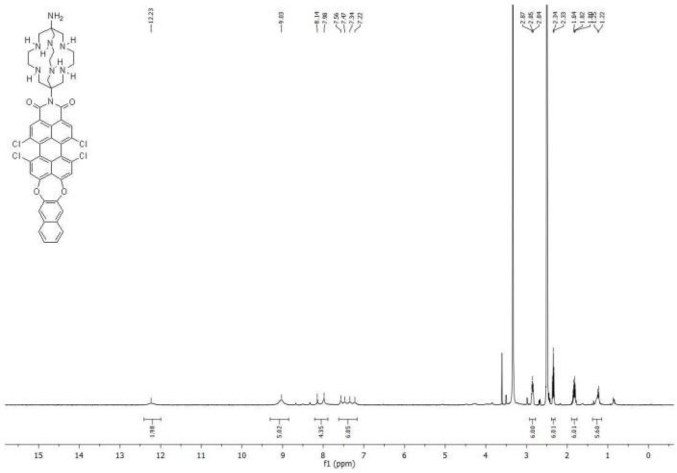

Figure S11. ^{1}H NMR spectrum of **1c**.

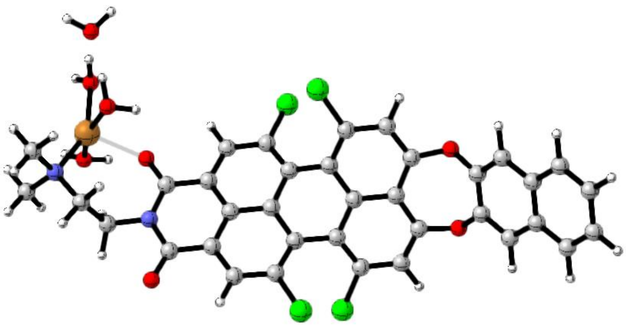

Figure S12. Optimized structure for the complex of **1b** with hydrated Cu^{2+} ion from B3LYP/6-31G(d,p) computations.

Table S1. Cartesian coordinates, electronic energies in Hartree, and the number of imaginary frequencies for the B3LYP/6-31G(d,p) fully optimized geometries of the ground state for dyes **1a-c** in methanol and complexes of dyes **1b,c** with Cu^{2+} ions.

1a
0 1
C,0,-2.3169462035,2.1471658435,-1.2404021342
C,0,-0.9199694632,2.236250638,-1.3474118079
C,0,-0.0708171039,1.2817391308,-0.764580121
C,0,-0.6897322109,0.0679458652,-0.3226040879
C,0,-2.0947183854,0.014948447,-0.1287911551
C,0,-2.9021980712,1.0839773224,-0.5778312597
C,0,0.1103759905,-1.0949122402,-0.0843052847
C,0,-0.4790875951,-2.115373165,0.6781601766
C,0,-1.8540956964,-2.1278830972,0.9605047946
C,0,-2.6703057764,-1.1078122576,0.5073704969
C,0,1.3815421745,1.3858340276,-0.5840111442
C,0,2.1495711423,0.1776340922,-0.6904295046
C,0,1.4669535353,-1.0859506501,-0.6423825583
C,0,2.091852969,2.555150901,-0.2877392222
C,0,3.4898459557,2.6062131645,-0.3512738265
C,0,4.2155549326,1.4851137321,-0.6959452151
C,0,3.5718334752,0.2266839172,-0.866753986
C,0,4.229772231,-0.9744642931,-1.2606871473
C,0,3.5132753127,-2.1286989287,-1.4871586067
C,0,2.1458938984,-2.1833370821,-1.1792175059
C,0,-4.1289193119,-1.1712095591,0.7405311474

N,0,-4.8893269816,-0.0855045191,0.2647382291
C,0,-4.3690002786,1.0430804037,-0.3978920579
O,0,-4.6698205245,-2.1073538058,1.3165945679
O,0,-5.1124781506,1.9321267935,-0.7950771852
C,0,-6.3263735348,-0.1322561394,0.4768579951
C,0,-6.8522600317,0.4125203058,1.6629369475
C,0,-8.2401301519,0.3568537597,1.8432231259
C,0,-9.0680661538,-0.2171040495,0.8821186787
C,0,-8.5200107501,-0.7485137189,-0.2823025143
C,0,-7.1384740561,-0.7187232274,-0.5112035318
C,0,-5.9750679107,1.0494482042,2.7357707847
C,0,-6.0669224054,0.2804844833,4.0675176302
C,0,-6.3077480692,2.5421641937,2.9214881765
C,0,-6.572359083,-1.306998658,-1.7993533652
C,0,-7.0751029284,-0.5366737185,-3.0353223558
C,0,-6.8734912971,-2.8134587573,-1.9127677595
H,0,-2.9446156383,2.9211378788,-1.664909805
H,0,-2.2864535097,-2.9467230405,1.5224281603
H,0,4.0167963758,3.5269557561,-0.1369056906
H,0,4.0245183223,-2.9988420504,-1.8782989093
H,0,-8.67700915,0.7687719068,2.7478236687
H,0,-10.1420087839,-0.2500940336,1.0407114743
H,0,-9.1739815527,-1.1933112412,-1.0261724885
H,0,-4.9347178876,0.9922772608,2.4043288185
H,0,-5.3879313093,0.7183519217,4.806812314
H,0,-5.7929240407,-0.7700096431,3.9312071703
H,0,-7.0795388971,0.3155417581,4.4832192085
H,0,-5.6310636445,2.9940490521,3.6544286433
H,0,-7.3321625936,2.6839147432,3.2819073836
H,0,-6.2026420007,3.0864990075,1.9780559906
H,0,-5.4846251146,-1.1988825595,-1.7749415423
H,0,-6.6157671224,-0.9363626479,-3.9456148711
H,0,-6.8226729805,0.5254194662,-2.9621667923
H,0,-8.161571467,-0.6203988994,-3.145366491
H,0,-6.4076829774,-3.2268608506,-2.8135433264
H,0,-7.9498168593,-3.0051233128,-1.9769085155
H,0,-6.4842467581,-3.3570569274,-1.046515075
Cl,0,0.4713029248,-3.3792091764,1.4401842637
Cl,0,-0.3132003354,3.5349521846,-2.3607451973
Cl,0,1.3284120679,-3.6672560408,-1.6213528254
Cl,0,1.3119339393,3.9959645754,0.3320546192
O,0,5.5594269727,1.68996653,-0.8704894148
O,0,5.5691517901,-1.0414453677,-1.5543475591
C,0,6.4922267518,0.8298097328,-0.3112810051
C,0,6.5003337578,-0.5423800027,-0.6561792959
C,0,7.4633303119,1.3508191707,0.5071130931
C,0,7.478309837,-1.3780780348,-0.1777040517
H,0,7.4406476661,2.408321076,0.7489720804
H,0,7.4665184805,-2.4248535813,-0.4631536098
C,0,8.4933657345,0.5172936547,1.0183367027
C,0,9.5160530755,1.0198863386,1.8676374506
C,0,8.5014032296,-0.8739582383,0.6688194179
C,0,10.4999816342,0.1856227057,2.3495660872
H,0,9.5078149295,2.0738240904,2.1305094916
C,0,9.5317314264,-1.7070499,1.1826349621
C,0,10.5078764258,-1.1889001259,2.0043012413
H,0,11.2763488543,0.5798304206,2.9979965981
H,0,9.5356375317,-2.7601475419,0.9163685351
H,0,11.2902230917,-1.8343023736,2.3916036766

Nimag=0 HF=-3889.4892118

1b

0 1

C,0,-2.7144344723,-2.5721664601,0.5144478664
C,0,-0.7941568742,-1.3054276277,-0.3396194346

C,0,-1.6668346783,-0.1809258407,-0.4905024463
C,0,-3.0694453708,-0.3494476208,-0.3590434284
C,0,-3.5850432521,-1.567616778,0.1348728022
C,0,-1.123570118,1.1112230826,-0.7843317277
C,0,-3.4115180147,1.8624549765,-1.2599711081
C,0,-3.938949944,0.6995127541,-0.7302280312
C,0,0.5755510112,-1.1466706742,-0.8407850396
C,0,1.1670524928,0.1589461484,-0.7356829376
C,0,0.3114941272,1.2949381377,-0.5382680591
C,0,2.7166635673,-1.954546684,-1.7079319755
C,0,3.3410929183,-0.7837790258,-1.3394281944
C,0,2.5869681445,0.322617307,-0.8516855896
C,0,3.1335185908,1.5973252126,-0.5298367887
C,0,2.3190661516,2.6248374978,-0.1023097251
C,0,-5.4057800212,0.5338444458,-0.6200547845
N,0,-5.8645481356,-0.6767405619,-0.0789703455
C,0,-5.0437151853,-1.7469115599,0.3014299728
O,0,-6.1884774044,1.4020545902,-0.9930506676
O,0,-5.5326472553,-2.7788575055,0.7529299499
C,0,-7.5790409979,0.635262647,3.5656225413
C,0,-8.3889695009,1.6793726406,1.5579614198
H,0,-3.1052063903,-3.4725107825,0.972448779
H,0,-4.0793247089,2.6300901924,-1.631321327
H,0,3.3006703108,-2.742894076,-2.1650046248
H,0,2.7715330548,3.5522794322,0.2240551361
H,0,-7.3175808234,1.6222800187,3.9597682089
H,0,-6.8667733154,-0.0919934181,3.9673748038
H,0,-8.5844312673,0.3751878054,3.9478169299
H,0,-8.1408812672,2.6484072157,2.0020116669
H,0,-9.4576741415,1.4756586992,1.7612097218
H,0,-8.2469802724,1.7639681884,0.4788456761
O,0,4.690524929,-0.7281250243,-1.5874665317
O,0,4.4646048455,1.9095436119,-0.6301910568
C,0,5.5546767895,-0.2641754623,-0.6073707598
C,0,6.5739940147,-1.0772402209,-0.1787797244
C,0,5.4376564034,1.0608202672,-0.1248838734
H,0,6.6460505908,-2.0867621839,-0.5700041457
C,0,6.3423665567,1.5577557769,0.7800396642
C,0,7.4122417065,0.7468431379,1.2436511779
H,0,6.236713303,2.5802165565,1.1275108995
C,0,7.5308298047,-0.5964258347,0.7543381383
C,0,8.6009380663,-1.4071034911,1.2204739707
C,0,9.5106557084,-0.9130786765,2.1286639141
H,0,8.6884885895,-2.4238403136,0.8483187533
C,0,8.3685363624,1.2256579285,2.1796265695
C,0,9.3934975725,0.4139924201,2.6121341316
H,0,10.3241982192,-1.5411214151,2.4784245398
H,0,8.2765693869,2.2432003607,2.548569509
H,0,10.1184228638,0.7897255434,3.3276209853
C,0,-7.3185055061,-0.8533444373,0.0989303034
H,0,-7.8040196837,-0.1468673933,-0.5709395014
H,0,-7.5676105806,-1.8672419076,-0.2178570703
C,0,-7.772245581,-0.6710696076,1.5530167314
H,0,-7.2469766,-1.4091546189,2.1651375521
H,0,-8.8487502269,-0.9304221656,1.5948892193
N,0,-7.5039867212,0.6542456261,2.1068867555
C,0,-2.0214967103,2.0590509593,-1.3005476812
C,0,-1.3348564152,-2.4370265115,0.2904146299
C,0,1.3477159581,-2.1337359825,-1.459217926
C,0,0.9267912976,2.4727290485,-0.0986745104
Cl,0,0.0269512686,3.7863482595,0.6318221908
Cl,0,-1.4791597266,3.4954743494,-2.1523555473
Cl,0,0.6534250629,-3.6169695429,-2.0794272584
Cl,0,-0.3224752566,-3.7065145036,0.9584282916
Nimag=0 -3635.1342255

1c
0 1
C,0,0.0319823146,-1.9809463406,-0.9389831803
C,0,1.4070121886,-1.7688581938,-1.1390309082
C,0,2.0289979825,-0.5572800332,-0.8008764894
C,0,1.2641291865,0.3466665704,0.0017692543
C,0,-0.140401622,0.1856404483,0.0981111977
C,0,-0.7538532452,-0.9828900123,-0.393249064
C,0,1.9124721172,1.4106095795,0.7063389345
C,0,1.0823537012,2.4498706788,1.1587447349
C,0,-0.3172451562,2.3272334079,1.1851202887
C,0,-0.9259512298,1.1788380628,0.7133230793
C,0,3.384590874,-0.1260337717,-1.1600438884
C,0,4.1010695804,0.6620699478,-0.1946692495
C,0,3.3685612659,1.3073351717,0.8593965508
C,0,4.0300555182,-0.3635061204,-2.3763043497
C,0,5.3961259754,-0.0981484868,-2.5535301105
C,0,6.1446968676,0.4066086579,-1.5139366286
C,0,5.5231479721,0.8163437899,-0.2988046222
C,0,6.2042436786,1.4211856186,0.7955876987
C,0,5.5137925718,1.8183233843,1.9212125374
C,0,4.1150848517,1.7533039827,1.9562209045
C,0,-2.3967541188,1.0251411248,0.7922977948
N,0,-2.9885448085,-0.0523830062,0.0977214232
C,0,-2.2186631568,-1.1797360625,-0.2511649298
O,0,-3.0756246051,1.8314359936,1.4221496722
O,0,-2.708645915,-2.2862065035,-0.4495630938
H,0,-0.4205041247,-2.920480939,-1.2308511673
H,0,-0.9238411269,3.1324004865,1.5807602148
H,0,5.8805896123,-0.3126837549,-3.4973342468
H,0,6.0684181034,2.1869354576,2.7743927195
Cl,0,2.3182021986,-3.1547376021,-1.7154173968
Cl,0,3.1702602393,-0.8952972959,-3.8063960502
Cl,0,3.3832007675,2.1166002643,3.5059798007
Cl,0,1.7150697802,4.0280497867,1.5979098508
O,0,7.4796606034,0.5905386865,-1.7792553193
O,0,7.5525928295,1.6685570378,0.8191783943
C,0,8.4310249073,0.1554232214,-0.8693950723
C,0,9.3880600261,-0.7378083058,-1.2816044984
C,0,8.4645039819,0.6963237152,0.4376525459
H,0,9.343934359,-1.1324499836,-2.2913543337
C,0,9.4560350279,0.3386858367,1.3170785066
C,0,10.4658042521,-0.579736409,0.9243722336
H,0,9.4652611012,0.7746286642,2.3106958393
C,0,10.4315421593,-1.1282552915,-0.4006973525
C,0,11.4416647501,-2.0483204883,-0.7915847065
C,0,12.4384151405,-2.41008356,0.0870791616
H,0,11.4136156019,-2.4617432071,-1.7957283849
C,0,11.5090688026,-0.9732869265,1.8055436017
C,0,12.4723797456,-1.868180698,1.3961735092
H,0,13.2050290996,-3.1141072158,-0.2215990168
H,0,11.5332517671,-0.5559913467,2.8081132974
H,0,13.264763662,-2.1622936887,2.0775373731
N,0,-11.6574149892,-0.5838198981,0.7195323863
N,0,-5.8331956083,0.7540542113,2.0726004902
N,0,-8.200886586,-0.7621868026,2.0924815604
N,0,-5.9895764634,1.4358472467,-1.5481578211
N,0,-8.3721681065,1.1818065179,0.007067056
N,0,-5.5051732147,-2.3674892891,-0.4943386851
N,0,-8.3013121214,-1.6746523565,-0.526563513
C,0,-4.5244335362,-0.0354038654,0.0045899505
C,0,-10.1846802702,-0.464566259,0.6361986282
C,0,-5.0760101556,-0.3285595016,1.4526238822
C,0,-6.4308863828,0.4269154825,3.37645646
C,0,-7.4704547446,-0.7282381326,3.3438333645

C,0,-9.6417429899,-0.9188716084,2.0224332564
C,0,-4.8881533876,1.4149213938,-0.5945355324
C,0,-6.9395806342,2.5473197358,-1.5147620335
C,0,-7.9015224506,2.526255362,-0.3066474958
C,0,-9.7828798144,1.0140412881,0.3397601159
C,0,-5.0636349628,-1.0898766634,-1.030649393
C,0,-6.4345662728,-3.1404930544,-1.3247442054
C,0,-7.7840740508,-2.4524619528,-1.6482235783
C,0,-9.7444204036,-1.4216323044,-0.5114388571
H,0,-10.105728288,-0.2821408967,2.7886310995
H,0,-10.0020113713,-1.9478763728,2.2088177323
H,0,-10.2706717416,-2.3743826083,-0.3704597434
H,0,-10.1161027982,-0.9935506917,-1.4642127803
H,0,-10.3853300163,1.3529929071,-0.515428806
H,0,-10.1110920441,1.6226553103,1.2082097194
H,0,-5.1362724204,2.0773443761,0.2278065311
H,0,-3.9721812151,1.8041252632,-1.0555654414
H,0,-5.9449067993,-0.5994884772,-1.440696042
H,0,-4.3449396347,-1.1754302984,-1.8604191729
H,0,-4.2221711677,-0.6497980108,2.0727850205
H,0,-5.7225990427,-1.2000257131,1.3568746382
H,0,-6.9285799307,1.3430748596,3.7164130528
H,0,-5.6732618674,0.1738355021,4.1420462397
H,0,-6.9595707532,-1.684097868,3.5397944426
H,0,-8.1586431992,-0.579310428,4.1848468635
H,0,-8.74489517,3.1845681457,-0.5531377748
H,0,-7.4067560947,2.9801341816,0.5655374265
H,0,-7.5352294022,2.452441174,-2.4300460451
H,0,-6.4572776094,3.5420051559,-1.5530777498
H,0,-5.9965578008,-3.4574655747,-2.2906858303
H,0,-6.6421134452,-4.0586579023,-0.7621737659
H,0,-8.4964092795,-3.247362646,-1.9018854902
H,0,-7.6847868786,-1.8372931344,-2.5598286718
H,0,-7.8671320829,-0.7509379871,-0.5579479649
H,0,-7.7849142921,-1.3970990674,1.4167398501
H,0,-7.821659006,0.8175678455,0.7880129886
H,0,-4.6903980294,-2.9354938188,-0.2879831368
H,0,-5.6400912032,1.3286573478,-2.4933903279
H,0,-5.1965027113,1.5335634998,2.2177743824
H,0,-12.0604150298,-0.3321472097,-0.1829830835
H,0,-12.0062375851,0.1208562549,1.3693447444
Nimag=0 HF=-4357.8942489

Complex of 1b with Cu^{2+}
+2 2
C,0,-1.8383471153,-2.8298566886,0.5507284544
C,0,-0.009530147,-1.5093690481,-0.4091130596
C,0,-0.9356658854,-0.4297426816,-0.5681618817
C,0,-2.3245816849,-0.6537731533,-0.3836989713
C,0,-2.7669736146,-1.8764042412,0.1654789464
C,0,-0.4587658192,0.8753704088,-0.9166462794
C,0,-2.7877270919,1.5240337287,-1.3311023522
C,0,-3.2482223185,0.3523176618,-0.7494324289
C,0,1.3341724834,-1.3084995798,-0.9549234798
C,0,1.868548371,0.0237752179,-0.9116609404
C,0,0.9704574467,1.1283052352,-0.7263767649
C,0,3.4807666631,-2.0544417066,-1.8619924304
C,0,4.0637054935,-0.8443890817,-1.5522388091
C,0,3.2758823843,0.2444737616,-1.0780367058
C,0,3.7747144629,1.553140918,-0.8212317737
C,0,2.927518263,2.5621654302,-0.4076352257
C,0,-4.6817562021,0.1250738554,-0.5643516823
N,0,-5.0844385929,-1.0873525028,-0.0406269826
C,0,-4.2012127168,-2.1201033255,0.3795205871
O,0,-5.5296515617,0.9936093895,-0.8620335509

O,0,-4.6484739925,-3.1395569992,0.8847325282
C,0,-8.7426692994,0.9812518252,2.1900890567
C,0,-9.4229646744,-0.4092389311,0.3393127475
H,0,-2.1739660266,-3.7292064398,1.0524928297
H,0,-3.4957247845,2.2547572573,-1.7022069793
H,0,4.0853827689,-2.8306001128,-2.3130011151
H,0,3.3497552741,3.5185018917,-0.1276242213
H,0,-9.5345507277,1.688694801,1.939037416
H,0,-7.9170528876,1.5112817442,2.6602635545
H,0,-9.1420162469,0.2344782827,2.8845049139
H,0,-10.2077126368,0.3093261926,0.0961410522
H,0,-9.818283992,-1.1374140424,1.0556024506
H,0,-9.1235656329,-0.9258717502,-0.5699504217
O,0,5.3980866002,-0.7407766503,-1.8467049605
O,0,5.0836121968,1.9203215513,-0.9691150486
C,0,6.2757702095,-0.2005479569,-0.91786996
C,0,7.3468605985,-0.950810487,-0.5017052253
C,0,6.1148600633,1.1333523483,-0.4767734059
H,0,7.4507374128,-1.9694137648,-0.8607566576
C,0,7.028109973,1.7039287885,0.374313222
C,0,8.1511627592,0.9596920143,0.8236738258
H,0,6.8871209981,2.7324040099,0.6899472024
C,0,8.3140112979,-0.3937422981,0.3767489485
C,0,9.4372875248,-1.1378111368,0.8286101609
C,0,10.3559499952,-0.5702787006,1.6832008037
H,0,9.5586029183,-2.1623155094,0.4887514073
C,0,9.1182902995,1.5152080203,1.7044834427
C,0,10.1952237691,0.766827391,2.1245382792
H,0,11.2102952202,-1.1476363288,2.0227481952
H,0,8.9933983853,2.5403520819,2.0411483879
H,0,10.9281335514,1.2006543448,2.7977237036
C,0,-6.530632725,-1.3426664286,0.0814083911
H,0,-6.9950770642,-1.0252922389,-0.8510339467
H,0,-6.6365386734,-2.4209228275,0.1654661493
C,0,-7.1751389178,-0.6475454964,1.3017828095
H,0,-6.4204407603,-0.0891376672,1.8589626603
H,0,-7.5936672965,-1.3969733226,1.9831412904
N,0,-8.2663290551,0.3130219748,0.9428980787
C,0,-1.4131127627,1.7720328168,-1.4334808641
C,0,-0.4767638339,-2.6433581363,0.2786512397
C,0,2.1314352884,-2.2846239208,-1.5633253459
C,0,1.5464553668,2.3508565155,-0.3528042
Cl,0,0.6119990337,3.6466846354,0.3613806944
Cl,0,-0.9584996031,3.1900560672,-2.3597945688
Cl,0,1.4826545704,-3.8138465424,-2.1107003915
Cl,0,0.6137490769,-3.8360099209,0.959181675
Cu,0,-7.5532655416,1.7449226679,-0.2820109776
O,0,-6.5001619449,2.6486887154,1.1476966788
H,0,-5.5600440815,2.5985869031,0.91391781
H,0,-6.7118020444,3.6086927624,1.2693222327
O,0,-7.3205635427,5.1920897268,0.8800155283
H,0,-6.6697954047,5.9040519174,0.7903400699
H,0,-8.039524438,5.5497127225,1.4216871664
O,0,-8.0793438081,0.8454151573,-1.9939852149
H,0,-7.3736609217,0.8943429261,-2.6577234595
H,0,-8.9180905307,0.9955636611,-2.455741351
O,0,-7.7780204113,3.5193133725,-1.1601285153
H,0,-7.7265191534,4.3014854449,-0.5585337777
H,0,-8.5008894132,3.6636515198,-1.786247874
Nimag=0 HF= -5580.9447123

Complex of 1c with Cu^{2+} ground state S_0
+2 2
C,0,-0.5163275447,-2.0819995769,-0.4138831176
C,0,-1.8783323821,-2.0871687992,-0.1812738745

C,0,-2.5548584589,-0.9864450996,0.4134663477
C,0,-1.8191588615,0.2357571289,0.503179471
C,0,-0.3967628031,0.2192055782,0.3742746769
C,0,0.2538372807,-0.9582156734,-0.0551168977
C,0,-2.5022076,1.4732957836,0.7127446242
C,0,-1.7029156255,2.5668791671,1.1480862471
C,0,-0.32279591,2.5440747849,1.0881355743
C,0,0.3437792952,1.3911723852,0.6315030712
C,0,-3.9290952004,-0.9636412887,0.8939823369
C,0,-4.6655234151,0.2652152514,0.7396434051
C,0,-3.9401513974,1.482177584,0.4818963131
C,0,-4.6041799121,-2.0203437477,1.5232626638
C,0,-5.9878399828,-1.9962312788,1.7329991767
C,0,-6.7312652261,-0.9033832434,1.3430505488
C,0,-6.0989229757,0.2756206343,0.8570500915
C,0,-6.7805629584,1.4749073431,0.5109663093
C,0,-6.0911260271,2.5699324368,0.0348712791
C,0,-4.6931423102,2.5677253727,0.0042096382
C,0,1.7858492354,1.4146349389,0.4673075635
N,0,2.4232492403,0.1603272695,0.2228756583
C,0,1.6895895705,-0.9821607879,-0.2293734126
O,0,2.441152767,2.4609726496,0.5434878091
O,0,2.2893598064,-1.947325043,-0.7335818212
H,0,-0.0309908183,-2.9288681344,-0.8813850358
H,0,0.2586765166,3.3959508115,1.416863781
H,0,-6.4910976363,-2.838406826,2.1913531145
H,0,-6.6488273119,3.4301541662,-0.3137175371
Cl,0,-2.780332714,-3.4558214309,-0.8512987946
Cl,0,-3.7441624543,-3.3863523545,2.2303086959
Cl,0,-3.9480516038,3.9251382709,-0.838337064
Cl,0,-2.4252985878,3.9482407999,1.9892366134
O,0,-8.0943787384,-0.998154365,1.5639192259
O,0,-8.1482500252,1.6393641911,0.6388591773
C,0,-8.9748302466,-0.6384822254,0.5602629504
C,0,-9.8805053789,-1.559528395,0.094189742
C,0,-9.0031110622,0.6995461767,0.092518487
H,0,-9.8451141711,-2.5757170247,0.4735257683
C,0,-9.9363211779,1.0961937918,-0.8332393246
C,0,-10.890500183,0.1708427946,-1.3345325311
H,0,-9.9435809676,2.1282209107,-1.1689129186
C,0,-10.8619649339,-1.1833061623,-0.8617852714
C,0,-11.8150663645,-2.108373871,-1.3677208688
C,0,-12.7516183244,-1.7144482406,-2.2975986349
H,0,-11.7916036733,-3.133326141,-1.0082939734
C,0,-11.870694468,0.5445439301,-2.2934667575
C,0,-12.7797718908,-0.3766529261,-2.7643899667
H,0,-13.4745396239,-2.4301196606,-2.6770962042
H,0,-11.8900131928,1.5707845081,-2.6495152303
H,0,-13.5239514112,-0.0797299674,-3.4970956041
N,0,11.4708641037,-0.3288217278,-0.7642963959
N,0,5.6806923694,0.4433368026,-1.6093457669
N,0,7.9299192026,-1.1889812653,-1.8129173597
N,0,6.0725543771,0.8985003402,1.2082479422
N,0,8.1434914863,1.4593815412,-0.555590253
N,0,5.6515280512,-1.7664007107,0.6458555816
N,0,8.302054301,-1.4878263577,0.7939401817
C,0,3.9210311599,0.0442947162,0.1624687499
C,0,10.023419052,-0.236738055,-0.5631258113
C,0,4.3103292588,-0.0704186679,-1.3496776123
C,0,6.2303465477,0.2207101284,-2.9878620207
C,0,7.0922621383,-1.0365474445,-3.0511287921
C,0,9.3831388345,-0.7876422994,-1.8845645564
C,0,4.6491775766,1.2478737941,0.8515992515
C,0,6.9256580866,2.0977233882,1.4865395108
C,0,7.5369608915,2.6034724559,0.1927228222
C,0,9.5930334794,1.2341880082,-0.3180980042

C,0,4.3168654237,-1.2080719504,1.0119120648
C,0,6.3508926406,-2.474326043,1.7828703878
C,0,7.7061240834,-1.8288999875,2.1054696824
C,0,9.7274373047,-1.0886582976,0.7061421396
H,0,9.4891669452,-0.0281430694,-2.6633835443
H,0,9.9660640494,-1.6559773019,-2.1973397675
H,0,10.3517330197,-1.9850537256,0.6817122677
H,0,9.9892526787,-0.513504361,1.5967924302
H,0,9.8317440442,1.5254147044,0.7063931838
H,0,10.1551220232,1.889827986,-0.9895632492
H,0,4.6565883565,2.1405295328,0.2341282141
H,0,4.1362830825,1.5120070092,1.7743039012
H,0,4.3312591612,-0.9239215465,2.0656632348
H,0,3.5827966338,-1.9977010614,0.8887289026
H,0,3.6012417972,0.5243035537,-1.9309876049
H,0,4.2302800096,-1.1005163742,-1.6893965811
H,0,6.8113853124,1.1060637808,-3.2544053526
H,0,5.4140126463,0.1518016948,-3.7113529881
H,0,6.4608601808,-1.9252332209,-3.106943129
H,0,7.7202247267,-1.0209815598,-3.9449231401
H,0,8.2940257788,3.3645626138,0.3968726016
H,0,6.7853164695,3.0702728968,-0.4471461437
H,0,7.7084306071,1.7845909899,2.1807090306
H,0,6.3396370985,2.8806628124,1.9744678561
H,0,5.7113227325,-2.482903914,2.6657706682
H,0,6.5060592144,-3.5118777136,1.4815250268
H,0,8.3328002618,-2.5389696814,2.6525038635
H,0,7.6261084437,-0.9269445066,2.715032235
H,0,8.2386277897,-2.3536616759,0.2627245884
H,0,7.9285520321,-2.1899504325,-1.6393817499
H,0,8.0454036358,1.6658682134,-1.5461258363
H,0,5.4864943803,-2.4605829989,-0.0804283454
H,0,6.0378636981,0.3738987801,2.0787002769
H,0,5.6249618931,1.4517374482,-1.4956712756
H,0,11.9648393903,0.0139146,0.0577989519
H,0,11.7621217624,0.26722633,-1.5369197539
Cu,0,6.9802691119,-0.2778563808,-0.2186316428
Nimag=0 HF=-5998.0217571

Table S2. Cartesian coordinates, electronic energies in Hartree, and the number of imaginary frequencies for the B3LYP/6-31G(d,p) fully optimized geometry of the excited state for the complex of dye **1c** with Cu^{2+} ions.

Complex of 1c with Cu^{2+} ground state S_1
+2 2
C,0,-0.5307624793,-2.0666902787,-0.5938590033
C,0,-1.911403816,-2.0621228306,-0.3391011668
C,0,-2.5574900824,-0.9506106736,0.22706386
C,0,-1.8083739536,0.2676821459,0.2712320163
C,0,-0.3990077144,0.2333104445,0.1224194425
C,0,0.235306755,-0.9533404455,-0.2954820857
C,0,-2.4782834565,1.5174374719,0.4663100007
C,0,-1.6715651377,2.5990646628,0.8605916197
C,0,-0.2710691362,2.5484454445,0.7787752002
C,0,0.3637349342,1.3932815376,0.357200085
C,0,-3.9242873725,-0.8976578171,0.7545005502
C,0,-4.6526693626,0.3278932949,0.5724176904
C,0,-3.9294350941,1.5275911582,0.2561940714
C,0,-4.5717323245,-1.9109360437,1.4679862729
C,0,-5.9433131664,-1.8544092013,1.7525625849
C,0,-6.6976251588,-0.7846102929,1.3229264052
C,0,-6.0785139988,0.3520285944,0.7265201256
C,0,-6.7664124392,1.5339767437,0.3296926903
C,0,-6.0784592331,2.601822402,-0.208628652
C,0,-4.6795040865,2.5945785009,-0.2541760636
C,0,1.8336655724,1.3793097649,0.1937958645

N,0,2.4485466285,0.1100172331,0.0233810426
C,0,1.6987091667,-0.9904292128,-0.4751242229
O,0,2.4795252234,2.4179938211,0.2218057063
O,0,2.2581628881,-1.9422986549,-1.0097504832
H,0,-0.057430361,-2.9502320417,-1.0038665994
H,0,0.3170603559,3.4190238542,1.0414245749
H,0,-6.4284281567,-2.6606493629,2.2874050594
H,0,-6.6368509662,3.4468514091,-0.5900359522
Cl,0,-2.7962833827,-3.4746494263,-0.8871186667
Cl,0,-3.7080001967,-3.2639796574,2.1661147645
Cl,0,-3.9466821136,3.9245051277,-1.1263470641
Cl,0,-2.338735613,4.0405307557,1.6066488316
O,0,-8.0356532249,-0.8494692851,1.618575645
O,0,-8.1176144344,1.7169614907,0.4576406658
C,0,-8.978621524,-0.5502209474,0.6466417019
C,0,-9.9250117746,-1.4913869438,0.3269691988
C,0,-9.0166122209,0.737707211,0.0627764235
H,0,-9.8780351426,-2.4692601348,0.7946977488
C,0,-10.001607437,1.0703913114,-0.8335682516
C,0,-11.0003485084,0.1239042553,-1.1853367661
H,0,-10.0144237361,2.0680503144,-1.2600476657
C,0,-10.9617521062,-1.1821198959,-0.5931960448
C,0,-11.9613679977,-2.1284219679,-0.9465934721
C,0,-12.952086724,-1.7989697849,-1.8445552606
H,0,-11.9303253237,-3.1163982128,-0.496145455
C,0,-12.037078906,0.4314910219,-2.107428806
C,0,-12.9902405357,-0.5087018404,-2.4296414281
H,0,-13.7108712587,-2.5293846691,-2.1078445908
H,0,-12.0646138576,1.4213841497,-2.5537117152
H,0,-13.7779144745,-0.2634111471,-3.1353288349
N,0,11.5042185385,-0.2678459801,-0.5141047401
N,0,5.750498929,0.4480529084,-1.6631953264
N,0,8.0923804504,-1.0613095503,-1.8820033706
N,0,6.0453552822,0.7512267864,1.2001322224
N,0,8.2519254402,1.6688718262,-0.3731663072
N,0,5.5134298483,-1.9938869796,0.6440274971
N,0,8.2239773582,-1.5607042741,0.7468689252
C,0,3.9551532781,-0.0745741337,0.0573868491
C,0,10.0364586411,-0.1536350772,-0.418395958
C,0,4.440697373,-0.2110159473,-1.4374529559
C,0,6.3516727107,0.3287141031,-3.0248479414
C,0,7.293461523,-0.8740513093,-3.1309446565
C,0,9.4991346545,-0.5459670483,-1.8411455026
C,0,4.6550855774,1.1144380986,0.800791689
C,0,6.8568444789,1.9098204621,1.6717759794
C,0,7.5700967199,2.6255375172,0.5211590573
C,0,9.6498797276,1.3155048202,-0.0528000697
C,0,4.2234083157,-1.3520797343,0.9492302563
C,0,6.25938473,-2.6072144142,1.7726298752
C,0,7.6204002019,-1.9216987918,2.0486022961
C,0,9.6549036997,-1.1504861517,0.7248819651
H,0,9.5650754476,0.3289206077,-2.4942739619
H,0,10.171849898,-1.3049245375,-2.2495715251
H,0,10.2902971065,-2.03554213,0.6311949115
H,0,9.8909641489,-0.6757958441,1.6815300799
H,0,9.8217828036,1.4690930056,1.0171280435
H,0,10.3389796864,1.9848434686,-0.5827820821
H,0,4.6926064836,2.0162995083,0.1970140504
H,0,4.0808037439,1.3678007597,1.694594332
H,0,4.2167500846,-1.0328789836,1.9955249507
H,0,3.4111144775,-2.0657215057,0.8422740684
H,0,3.6874286119,0.2433819368,-2.0883013745
H,0,4.5229231304,-1.2594025026,-1.7185260978
H,0,6.9007081808,1.2556802613,-3.2053533749
H,0,5.5768104734,0.2604163509,-3.7954889932
H,0,6.7127021184,-1.7898654458,-3.2643364116

H,0,7.937928825,-0.7655676241,-4.0087622148
H,0,8.2580083988,3.3688632731,0.9437402921
H,0,6.8448237008,3.1813619467,-0.0805265007
H,0,7.5951143539,1.5113006056,2.3739348836
H,0,6.2379460553,2.6252324526,2.2250326537
H,0,5.6674307133,-2.6112421794,2.6932242327
H,0,6.4550636393,-3.6541307583,1.5227290959
H,0,8.2588173087,-2.6151364625,2.6077475217
H,0,7.5286487782,-1.0173028123,2.6557007104
H,0,8.1644992997,-2.4143887236,0.1955784586
H,0,8.1574157866,-2.0686200805,-1.7685691401
H,0,8.2447870047,2.0687331199,-1.3056830035
H,0,5.3468850809,-2.7159841658,-0.0477774305
H,0,5.9680428499,0.1289914095,1.9981532855
H,0,5.6086101045,1.4429007015,-1.5147384772
H,0,11.9260198203,0.0231735247,0.3674653778
H,0,11.8527856219,0.3927918107,-1.2075951938
Cu,0,7.0441727475,-0.294123284,-0.3082881515
Nimag=0 HF= -5998.0042041

Table S3. Cartesian coordinates, electronic energies in Hartree, and the number of imaginary frequencies for the optimized geometry of the excited state for dye **1b** in methanol at PBE1PBE/6-311+G(2d,p) level of theory.

1b
0 1
C,0,-3.1770820605,-2.6459133904,-0.6652081927
C,0,-1.0699513377,-1.4224193311,-0.6043631957
C,0,-1.809178907,-0.2268514806,-0.3951981868
C,0,-3.2199771184,-0.230823862,-0.5417856163
C,0,-3.8935258687,-1.4513770646,-0.7111211576
C,0,-1.1356326834,0.9734519484,-0.0414505954
C,0,-3.2152978025,2.1843232495,-0.4276829046
C,0,-3.9208072697,0.9860867377,-0.5236606658
C,0,0.3497993968,-1.2870385538,-0.7883596449
C,0,1.0142650565,-0.2183112453,-0.1007442957
C,0,0.2159654919,0.8466859377,0.4327485135
C,0,2.5153325998,-2.0375044678,-1.567845075
C,0,3.1498571695,-1.180489544,-0.6949204983
C,0,2.4347359448,-0.214049537,0.0478441579
C,0,2.9752593623,0.7571644633,0.9208445819
C,0,2.168974539,1.6114380193,1.641523034
C,0,-5.3649776072,1.0013926331,-0.6889175626
N,0,-5.9927078746,-0.2398669833,-0.8051368871
C,0,-5.3392112001,-1.4699779121,-0.8478747078
O,0,-6.0187575233,2.0393492172,-0.7364543618
O,0,-5.9877201654,-2.5034138979,-0.9929957205
C,0,-8.3525869907,-0.444375004,2.7047939386
C,0,-8.6444710248,1.4633111626,1.3056653903
H,0,-3.7157320661,-3.5851008193,-0.6617092796
H,0,-3.7458247753,3.1202296911,-0.5489554197
H,0,3.1196455905,-2.6746583307,-2.2005111382
H,0,2.6256063673,2.2529403497,2.3840737388
H,0,-8.1454478538,0.2188718358,3.5479561143
H,0,-7.7856792521,-1.367518878,2.8454571796
H,0,-9.4287089309,-0.6953730013,2.7269964855
H,0,-8.4522383656,2.0968613815,2.1747089277
H,0,-9.7381635117,1.3328071384,1.2121157842
H,0,-8.2744353799,1.9851609739,0.4227680009
O,0,4.5018176856,-1.3744837467,-0.6996204814
O,0,4.2956800248,0.9586029684,1.2046480543
C,0,5.4988305319,-0.7637635523,-0.0931359817
C,0,6.7517405502,-1.2815730214,-0.392132655
C,0,5.4002535748,0.3520217752,0.8200266519
H,0,6.7945956519,-2.1170148988,-1.080586492

C,0,6.5607536114,0.8744127879,1.373295569
C,0,7.8108814776,0.356479923,1.0752564978
H,0,6.4559907182,1.7095078229,2.0554855331
C,0,7.9097479297,-0.7592244693,0.160235141
C,0,9.1946231579,-1.2876886978,-0.1455916689
C,0,10.3063000112,-0.7483920239,0.4186952645
H,0,9.2663895599,-2.1233248036,-0.8323443087
C,0,9.0011869007,0.8890092914,1.6429352512
C,0,10.2083174877,0.3533968105,1.3241858757
H,0,11.2846502138,-1.1521701954,0.1856001027
H,0,8.9250277443,1.7246874251,2.3291768904
H,0,11.1146654663,0.7602961235,1.7572388102
C,0,-7.4465835123,-0.2642878693,-0.9358810533
H,0,-7.7512798297,0.7304668367,-1.252476855
H,0,-7.6987916506,-0.9766349158,-1.7219802181
C,0,-8.1578290708,-0.6915625021,0.3392468041
H,0,-7.7953495105,-1.6828829352,0.6188917453
H,0,-9.2327261992,-0.7983893044,0.0980611364
N,0,-7.9573637569,0.1977276268,1.4678740655
C,0,-1.8575704173,2.1774043508,-0.1999675757
C,0,-1.8018549781,-2.6309852237,-0.6038670156
C,0,1.1365892174,-2.0836055553,-1.6305859631
C,0,0.8069603142,1.649458079,1.4172814326
Cl,0,-0.1376580609,2.594153767,2.5219810243
Cl,0,-1.0296595468,3.7075477288,-0.3133519242
Cl,0,0.4471830099,-3.0298216998,-2.9091756689
Cl,0,-1.0068805454,-4.1571601082,-0.3234327992

Nimag=0 HF= -3633.0046098
